# Elongate dendritic phytoliths as indicators for cereal identification and domestication: exploring a 3D morphometric approach

**DOI:** 10.3389/fpls.2025.1643447

**Published:** 2025-10-06

**Authors:** Rosalie M. Hermans, Mao Li, William H. Brightly, Timothy J. Gallaher, Wouter Smagghe, Hannah Lee, Leticia Arco, Lara Stas, Perseverence Savieri, Luc Vrydaghs, Karin Nys, Christophe Snoeck, Caroline A. E. Strömberg

**Affiliations:** ^1^ Archaeology, Environmental Changes and Geo-Chemistry Research Group (AMGC), Vrije Universiteit Brussel, Brussels, Belgium; ^2^ Donald Danforth Plant Science Center, Saint Louis, MO, United States; ^3^ School of Biosciences, University of Sheffield, Sheffield, United Kingdom; ^4^ Department of Biology, University of Washington, Seattle, WA, United States; ^5^ Department of Plant Biotechnology and Bioinformatics, Ghent University, Ghent, Belgium; ^6^ Center for Plant Systems Biology, Vlaams Instituut voor Biotechnologie, Ghent, Belgium; ^7^ Artificial Intelligence Lab, Department of Computer Science, Vrije Universiteit Brussel, Brussels, Belgium; ^8^ Core Facility for Support for Quantitative and Qualitative Research (SQUARE), Vrije Universiteit Brussel, Brussels, Belgium; ^9^ Biostatistics and Medical Informatics Research Group (BISI), Vrije Universiteit Brussel, Brussels, Belgium; ^10^ Burke Museum of Natural History & Culture, University of Washington, Seattle, WA, United States

**Keywords:** phytoliths, Elongate dendritic, morphometrics, Pooideae, archaeology, cereals, persistent homology, machine learning

## Abstract

**Introduction:**

The phytolith (plant silica) morphotype, Elongate dendritic, is used to indicate the presence of domesticated grasses (cereals) from the Pooideae subfamily, such as wheat and barley, in the archaeological record, but related wild taxa also produce Elongate dendritic that closely resemble those of cereals. By examining the morphometric traits of Elongate dendritic in a diverse set of extant Pooideae taxa, we evaluate its effectiveness as a proxy for cereal domestication and identification.

**Methods:**

We investigated the occurrence of Elongate dendritic across a wide range of Pooideae taxa and generated 3D meshes of phytoliths using confocal microscopy. From these meshes, we extracted geometric morphometric and topological traits, which served as input for machine learning (ML) models to assess the taxonomic resolution of Elongate dendritic. Regression models and linear discriminant analyses (LDAs) were applied to test for links between morphometric traits, domestication status, and ploidy level.

**Results:**

Our results show that Elongate dendritic occurrence is likely an ancestral trait within Pooideae, with high levels largely confined to Triticeae (wheat, barley, rye) and Avena (oats). Machine learning applied to 3D phytolith traits captured meaningful taxonomic patterns, with more reliable identification at broader taxonomic levels than at finer ones. However, the approach requires further refinement before it can be robustly applied to archaeological samples. Regression models and LDA demonstrated that while domestication significantly influences morphometric variation, ploidy level does not, although further study is warranted.

**Discussion:**

These findings offer important guidance for archaeologists and biologists studying crop domestication. By integrating 3D morphometrics, topological data analysis, and ML, this study introduces a new approach to quantitative phytolith identification. Continued expansion of reference datasets, coupled with methodological refinement, will be essential for improving identification at finer taxonomic levels and unlocking the full potential of Elongate dendritic in the study of domestication and 168 cultivation practices.

## 1 Introduction

Cultivated crops have played a vital role in human history, and tracing the domestication of individual crops is essential for reconstructing ancient agricultural systems and dietary practices (Fuller et al., 2023; Zohary et al., 2012). Within the Poaceae subfamily Pooideae, wheat (*Triticum* spp.), barley (*Hordeum* spp.), rye (*Secale* spp.), and oats (*Avena* spp.) were among the earliest domesticated cereals. For example, archaeobotanical evidence indicates that wheat (*Triticum aestivum*) and barley (*Hordeum vulgare*) were domesticated in the Fertile Crescent by the 11^th^ millennium cal BP (Zohary et al., 2012). Archaeological plant remains provide a means for tracking the domestication of these cereals, including pre-domestication stages (Weiss et al., 2008), identifying the geographical origins of (multiple) domestication events (Nan et al., 2023; Willcox, 2005), tracing the spread of these crops in time and space (Zohary et al., 2012), assessing phenotypic divergence due to natural selection and domestication processes (Burger et al., 2007; Willcox, 2004), and reconstructing agricultural practices and economic uses in past societies (Devos et al., 2013; Harvey and Fuller, 2005; McClatchie et al., 2015).

**Figure 1:**
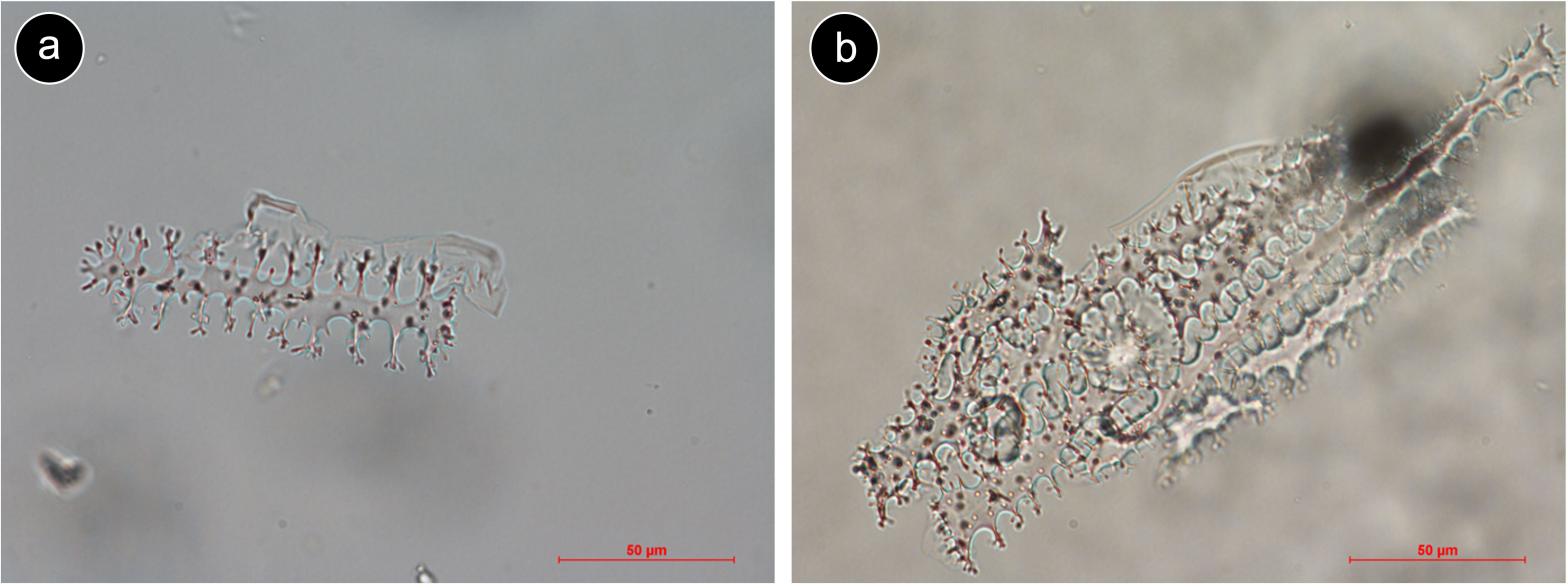
Elongate dendritic derived from inflorescence tissue from *Triticum aestivum*. **(a)** Isolated Elongate dendritic. **(b)** Articulated Elongate dendritic (silica skeleton). Source: Strömberg lab modern plant-based phytolith reference collection.

Microscopic plant silica bodies (phytoliths) are among the most commonly used tools to track domestication and cultivation of cereals (Ball et al., 2016; Harvey and Fuller, 2005). They form in many plants when dissolved silica in the groundwater is taken up by roots and precipitated in and around cells, and are often diagnostic of important taxonomic or ecological groupings of plants (Piperno, 1988). Pooideae grasses produce a distinctive phytolith morphotype, Elongate dendritic, in the long cells of inflorescence bracts (palea, lemma, and glume) (ICPT, 2019). Occurring either as isolated forms or in articulated groups (**Figure 1**) (Berlin et al., 2003; Helbaek, 1960; Rosen, 1992), these phytoliths are found in many domesticated cereals in the Pooideae and are therefore used in archaeology as a cereal marker, especially in contexts like cooking or storing vessels (Berlin et al., 2003; Wang et al., 2016).

Based on quantitative assessments, scholars have suggested that morphometric traits from Elongate dendritic may be taxonomically diagnostic at the genus or species level (Ball et al., 2017; Portillo et al., 2006). However, current analyses—both of isolated Elongate dendritic (Portillo et al., 2006) and of the spaces between individual dendritic processes (Ball et al., 2017; **Figure 1**)—rely exclusively on geometric morphometric descriptors (e.g., length, width, solidity, sphericity). These metrics are well suited for quantifying closed shapes but not branching structures. As a result, they may fail to capture the topological complexity of dendritic branching (Li et al., 2017), which may be critical for resolving fine-scale taxonomic differences among Elongate dendritic from closely related taxa.

Another potential problem with the common use of Elongate dendritic as indicators of cereals in archaeological assemblages is that they also occur in wild relatives and other Pooideae taxa—often referred to as ‘potential confusers’ (ICPT, 2019; Novello and Barboni, 2015; Parry and Smithson, 1966). Yet the limited morphometric sampling of Elongate dendritic in extant grasses prevents a clear understanding of their broader distribution and morphometric variability, leaving questions about the taxonomic resolution of Elongate dendritic.

Finally, it is unknown whether domestication or changes often associated with domestication (polyploidization) resulted in morphometric variation in Elongate dendritic. Whereas traits such as non-shattering spikes and larger seeds, referred to as the domestication syndrome, are well-documented in cereal macro remains (Purugganan and Fuller, 2009), potential changes at the microscopic level remain largely unexplored. Polyploidization is known to have occurred in many domesticated grasses, leading to larger cells to accommodate increased amounts of genetic material (Soltis et al., 2004). For example, in *Triticum*, the diameter and the number of pits in Papillate, another phytolith morphotype that occur in grass inflorescences increase with ploidy level, likely due to overall cell enlargement (Tubb et al., 1993). These results indicate that morphology may be useful in determining the ploidy level of wheat in archaeological records. Differentiation between taxa with different ploidy levels would undoubtedly be helpful in tracking the domestication of grasses by allowing distinction between different species (and their varieties), such as the free-threshing tetraploid *Triticum turgidum* and hexaploid *Triticum aestivum.*

In this study, we attempt to fill these knowledge gaps by first surveying the occurrence of Elongate dendritic across extant Pooideae species to identify the taxa that produce this morphotype. Second, focusing on the producing taxa, we move beyond 2D morphometric traits by developing a comprehensive 3D morphometric dataset spanning both cereal and non-cereal species within the Pooideae subfamily. Whereas 3D morphometric analysis is relatively new in phytolith research, it has been successfully applied to other morphotypes (Gallaher et al., 2020). In addition to the analysis of geometric morphometric traits, we apply persistent homology (PH) to capture topological traits. Topological traits describe how parts of a structure are connected and arranged. PH enables quantification of structural features such as branching features across multiple spatial scales (Li et al., 2017, 2019). These topological traits are particularly relevant for Elongate dendritic, whose taxonomically informative features may lie in their complex branching architecture. We employ these 3D approaches to test two hypotheses:

*H1. 3D morphometric traits of Elongate dendritic differ between wild and domesticated* sp*ecies, and among taxa with different ploidy levels (diploid, tetraploid, hexaploid).*

To test this hypothesis, we analyze the 3D geometric morphometric and topological traits using regression models and linear discriminant analyses.

*H2: 3D morphometric traits of Elongate dendritic can be used to distinguish taxa at the genus*, sp*ecies, and subspecies levels.*

Using the same morphometric dataset as in H1, we first assess the phylogenetic signal of morphometric traits to identify those with the strongest taxonomic structure. We then apply machine learning models to evaluate how effectively traits differentiate taxa across multiple levels of classification within Pooideae.

This study provides a broad-scale, quantitative evaluation of Elongate dendritic within Pooideae. By integrating 3D morphometrics, topological data analysis, and machine learning, it advances the methodological toolkit for phytolith analysis and challenges assumptions about their specificity to domesticated cereals—ultimately refining their utility in archaeological studies.

## 2 Materials and methods

### 2.1 Elongate dendritic occurrence

#### 2.1.1 Sample selection and chemical processing

To determine the occurrence of Elongate dendritic within the Pooideae, we conducted a survey of inflorescence samples. Elongate dendritic are characterized by branched processes along both long margins, and in some cases also at the ends. The degree of branching can vary within a single phytolith, ranging from strongly branched to dentate (ICPT, 2019). For the purposes of this study, elongated phytoliths were considered Elongate dendritic when they exhibited at least one branched process.

In total, 312 inflorescence samples were analyzed using transmitted light microscopy, comprising 230 samples processed for this study and 82 samples from the Strömberg Lab reference collection (UWBM). An additional 35 observations from the literature were incorporated (Parry and Smithson, 1966), resulting in a combined dataset of 347 samples, representing nine of the 16 Pooideae tribes, 55 genera, and 188 species (**Supplementary Table S1**). Taxonomic nomenclature follows Plants of the World Online (POWO, 2023).

Whole inflorescences were collected and processed to capture within-organ variability (Mulholland et al., 1990). Phytoliths were extracted using two protocols (**Supplementary Table S1**). Most samples (n = 244) were processed with wet oxidation, using 68% HNO₃ and KClO₃ for 4-6 hours to remove organics, followed by 37% HCl to eliminate carbonates. A subset of earlier samples (n = 68) was processed using a combined wet and dry oxidation method, involving ashing at 500°C for six hours, then treatment with 65% HNO₃ and 37% HCl (see Hermans et al. (2025) for details on both methods). The combined wet and dry oxidation method was initially used to assess Elongate dendritic occurrence across taxa in the Pooideae subfamily as it requires fewer chemicals, but we later adopted full wet oxidation to optimize 3D imaging, as it produces fewer silica skeletons and more isolated phytoliths, which are required for 3D imaging.

#### 2.1.2 Microscopy observations

Microscopic slides were systematically scanned at 200× magnification with plane-polarized light (PPL) using a Zeiss Axioscope 5 TL/RL polarizing microscope or a Nikon i80 compound microscope. All samples processed for this study yielded phytoliths.

We grouped samples into three categories based on an estimate of Elongate dendritic abundance: (1) ‘absent’ (no Elongate dendritic observed), (2) ‘low’ (<5% of total phytoliths), and (3) ‘high’ (≥5% of total phytoliths). The 5% cutoff was chosen as an arbitrary threshold to separate cases of very limited occurrence from those in which Elongate dendritic formed a consistently visible component. For samples with <5% abundance, Elongate dendritic typically numbered fewer than ten individuals per slide, while the same slides contained hundreds of other phytoliths, which is technically not ‘absent’. For the dataset of Parry and Smithson (1966), descriptions of “some Elongate dendriform” were classified as low producers, while “Elongate dendriform present” was classified as high producers in this study.

#### 2.1.3 Ancestral state reconstruction

To evaluate whether “high” Elongate dendritic occurrence represented an ancestral condition within Pooideae or was a derived trait restricted to specific lineages (e.g., domesticated cereals), we reconstructed ancestral states using Bayesian threshold models (Felsenstein, 2012) implemented in the R package phytools (Revell, 2024).

For these analyses, we first constructed a phylogenetic tree based on three plastid coding genes (matK, ndhF, and rbcL) retrieved from GenBank for each species (**Supplementary Table S1**). Sequences were aligned using Clustal Omega v1.2.22 (five refinement iterations), manually adjusted in Geneious Prime (Sievers et al., 2011), and analyzed in MrBayes v3.2.6 under the HKY85 substitution model, using *Zea* mays as the outgroup (Huelsenbeck and Ronquist, 2001; Ronquist and Huelsenbeck, 2003). Two independent runs of four heated Markov chains (ngen = 3,000,000, temp = 0.2) reached convergence (average standard deviation of split frequencies = 0.039).

The resulting consensus tree was converted to a chronogram using penalized likelihood in ape (Paradis and Schliep, 2018), selecting a correlated clock model with a maximum root age of 100 Ma and λ = 1 as the best fit (**Supplementary Data Sheet 1**). Low Elongate dendritic occurrence was modeled as intermediate between absence and high production, with species-level probabilities proportional to the relative number of individuals showing each state. We ran two chains of 400 million generations, discarding the first 80 million as burn-in. Convergence diagnostics (R̂ ≈ 1.0) and effective sample sizes (ESS > 200) indicated adequate sampling of the posterior distribution.

### 2.2 Confocal imaging and 3D morphometric data generation

#### 2.2.1 Sample selection and chemical processing

We selected 91 out of our 312 inflorescence samples containing Elongate dendritic for 3D morphometric analysis. This selection consisted of inflorescence samples from the genera *Hordeum*, *Secale*, *Avena*, and *Triticum*, alongside wild relatives from these genera and several other Pooideae taxa. For many taxa, multiple inflorescence samples were included (**Supplementary Table S1**). We selected samples with at least 20 Elongate dendritic. Specimens were selected with minimal attached silica or organic matter, since these could complicate image segmentation and 3D reconstruction. This selection ensured representativeness of Elongate dendritic across Pooideae taxa.

Phytoliths were pretreated for confocal microscopy using fluorescent staining. Following Gallaher et al. (2020), samples were treated in ethanol, sequentially cleaned with detergent and optical lens cleaner. Phytoliths were then stained with either fluorescein isothiocyanate (FITC) or rhodamine B isothiocyanate (RBITC) (Friedrichs, 2013) and mounted on microscope slides using a 70:30 Permount/xylenes solution.

#### 2.2.2 Confocal imaging and 3D surface mesh creation

**Figure 2:**
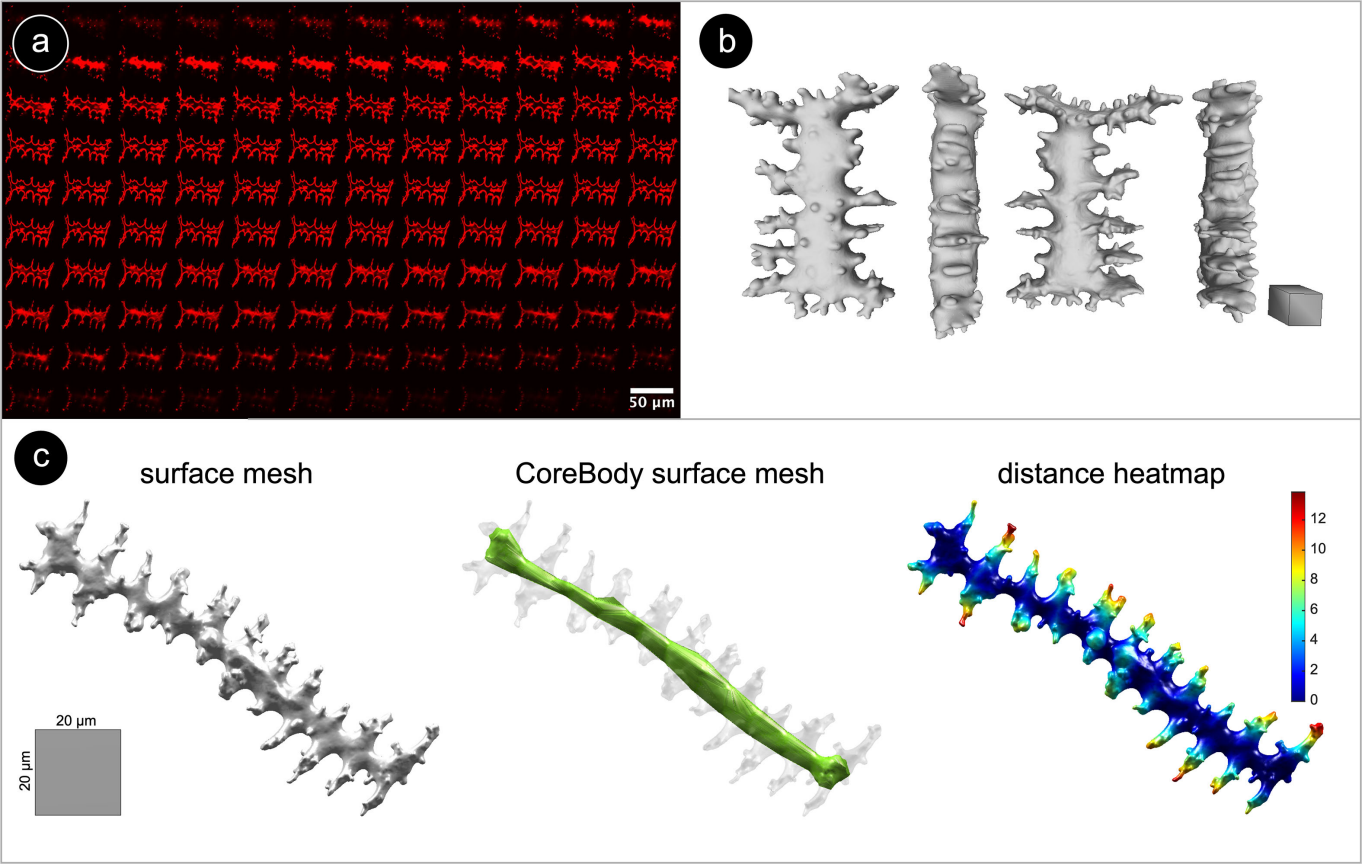
**(a)** Grid view of a fluorescent confocal Z-stack of Elongate dendritic from *Triticum aestivum* ssp. *sphaerococcum.* Phytoliths were stained with RBITC, resulting in a red fluorescent coating. **(b)** Surface mesh of an Elongate dendritic from *Triticum aestivum* ssp. *sphaerococcum* in different views. Scale: 20 µm cuboid. **(c)** Surface mesh, surface mesh of the ‘core body’, and a surface heatmap visualization of the shortest-path distance from the dendritic branches to the ‘core body’ for an Elongate dendritic from *Aegilops biuncialis*.

A Nikon A1R HD25 laser scanning confocal microscope with a Plan Apo λ 60× oil objective (NA = 1.4) was used to capture optical sections for each phytolith (**Figure 2a**). We used the resonant scanner, a 488 nm laser for FITC and a 561 nm laser for RBITC (~50% power), a substrate beam splitter, and a transmitted light detector (gain ~50 V). Z-stacks of entire phytoliths were acquired with a step size of 0.1–0.13 µm in the Z-direction using 8× frame averaging. Approximately 30 images per sample were captured, yielding on average 20–25 usable 3D surface meshes (**Supplementary Table S1**). This number of phytoliths per sample was chosen to ensure a large enough dataset across samples while remaining feasible within the practical constraints of 3D imaging and processing.

Image stack and 3D surface mesh processing were performed using ImageJ v1.53f (Schindelin et al., 2012), Imaris v9.9, and PyMeshLab (Cignoni et al., 2008). In FIJI, non-Elongate dendritic fluorescent signal (e.g. organic material, attached silica) was manually removed from images when necessary (Gallaher et al., 2020). 3D reconstructions of confocal z-stacks were generated in Imaris using the ‘surface creation’ tool (**Figure 2b**). To retain only the outer shell of the meshes required for further analysis, we applied some cleaning steps using PyMeshLab (ambient occlusion filtering, selecting faces by color, moving selected faces to a separate layer, deleting internal mesh elements, closing holes). Note that the 3D surface models are digital reconstructions of Elongate dendritic, and due to image processing and 3D modeling, dendritic branches may appear slightly less sharp than the original branches.

#### 2.2.3 Extraction of 3D morphometric traits

3D surface meshes were analyzed in MATLAB (R2017a, MathWorks). All scripts were made available in the GitHub repository. Each mesh was oriented by performing a principal component analysis (PCA) on the 3D coordinates of its surface vertices. The resulting three principal axes correspond to the phytolith’s ∼length (PC1), ∼width (PC2), and ∼height (PC3). For each mesh, we constructed its ‘core body’ as a separate mesh representing the internal structure of the phytolith (**Figure 2c**). For both the original and core body meshes, several geometric traits relating to size (e.g., volume, surface area) and shape (e.g., solidity, sphericity) were calculated (**Supplementary Table S2**).

**Figure 3:**
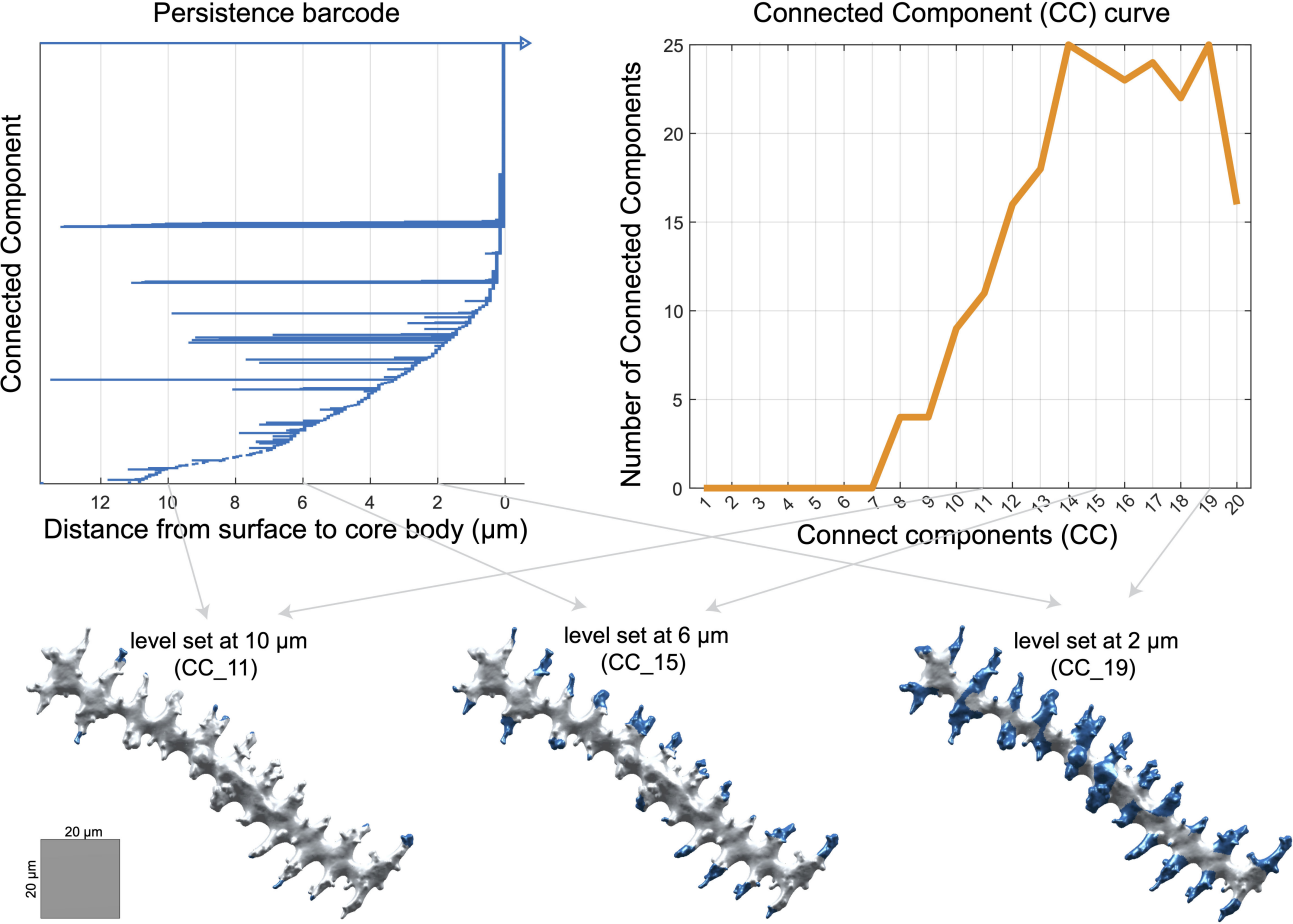
Persistence barcode, connected components (CC) curve, and visualization of persistence barcode values on a phytolith at different level sets. All numeric units represent measurements in µm. A value of 0 µm indicates that the shortest-path distance to the core body has reached zero.

Next, we used topological data analysis to quantify branching patterns with persistent homology (PH), which captures the connectivity of dendritic branches (Li et al., 2019). For each phytolith, we calculated the geodesic distance to its ‘core body’: branch tips had the largest values, while points near the ‘core body’ were close to zero. As the distance threshold decreased from maximum to zero, we recorded when branches appeared and when they merged with each other or the core. These events were summarized in persistence barcodes (**Figure 3**), generated with javaPlex (Adams et al., 2014), from which several branching traits were derived (**Supplementary Table S2**).

In addition to the branching traits derived directly from the persistence barcodes, we extracted two additional sets of topological traits. First, we quantified overall differences in branching architecture across phytoliths by calculating pairwise bottleneck distances between persistence barcodes using Dionysus (Morozov, 2007). The bottleneck distance measures how similar or different the branching architectures are between two phytoliths: a small distance indicates very similar branching, while a large distance reflects more divergent patterns. We applied multidimensional scaling (MDS) to the distance matrix and retained the first 20 MDS coordinates as persistent homology traits (PH1–PH20) (**Supplementary Table S2**).

Second, we calculated connected component traits (CC1–CC20) per phytolith to describe how many dendritic branches persist at different distances from the ‘core body’. We counted the number of branches at 1 µm intervals, starting 20 µm away from the core body and moving inward to 1 µm (**Figure 3**). CC1 corresponds to branches longer than 20 µm, CC2 to branches longer than 19 µm, and so on, with CC20 representing branches longer than 1 µm (**Figure 3**; **Supplementary Table S2**).

### 2.3 Phylogenetic signal in morphometric traits

We tested whether variation in Elongate dendritic morphometric traits reflected shared evolutionary history by assessing phylogenetic signal using both multivariate and univariate approaches. We estimated Pagel’s λ (Pagel, 1999) for each individual trait, except for the CC traits, which are highly interdependent and therefore better interpreted from the multivariate results. Univariate tests were conducted with phytools (Revell, 2024) using species means and standard errors, and significance was assessed against a null model assuming no phylogenetic structure.

Because fitted λ values alone are not reliable indicators of the strength of phylogenetic signal, we used multivariate effect sizes (Z) to compare across trait sets (Collyer et al., 2022). For the multivariate analysis, we used a matrix-based generalization of Blomberg’s κ (Blomberg et al., 2003; Mitteroecker et al, 2025), implemented in the R package geomorph (Baken et al., 2021; Adams et al., 2025). Analyses were performed separately for three trait sets: (1) geometric and branching traits, (2) PH1–PH20 traits, and (3) CC1–CC20 traits.

### 2.4 Effects of domestication and ploidy level on 3D morphometric traits

#### 2.4.1 Species-level analysis of mean morphometric traits

We assessed whether 3D morphometric traits of Elongate dendritic differed between wild and domesticated species and among taxa with different ploidy levels (diploid, tetraploid, hexaploid). All analyses were performed in R (R Core Team, 2024), with scripts archived in the Zenodo repository. Domestication status and ploidy information were compiled from published sources (**Supplementary Table S1**). Because some taxa occur in both wild and domesticated forms, and several species are mixed-ploidy (Kolář et al., 2017; Zohary et al., 2012), these classifications may introduce some noise into the analysis, and the results should be regarded as preliminary.

For each inflorescence sample (n = 91), morphometric trait values were averaged across approximately 20–25 phytoliths per sample. To avoid redundancy, traits with very strong correlations (|r| > 0.90) were grouped, and one representative trait from each group was retained for further analysis. We then performed a PCA with varimax rotation to facilitate interpretation of the components (Abdi and Williams, 2010). The rotated components (RCs) were selected using the elbow method, which identifies the point where adding more components contributes little additional variance (Jolliffe, 2002). These RCs were then used as predictors in regression models to examine their relationships with domestication status and ploidy level.

Domestication was modeled using logistic regression, with “domesticated” as the reference category and “wild” as the comparison. Ploidy level was modeled using ordinal logistic regression, treating diploid, tetraploid, and hexaploid states as ordered categories (Venables and Ripley, 2002). For components showing significant associations (p < 0.05), we visualized the distributions of the most influential morphometric traits (defined as those with high factor loadings (>0.60) on the RCs) using boxplots (Wickham, 2016).

#### 2.4.2 Analysis of individual phytolith morphometric traits

To evaluate how well individual phytolith morphometric traits related to domestication status and ploidy level, we performed linear discriminant analysis (LDA) on all individual phytolith traits (Venables and Ripley, 2002). The same preprocessing steps were applied as before, including correlation filtering and PCA with varimax rotation, and the resulting RCs were used as predictors in the LDAs. Group separation was visualized using scatterplots and density plots (Wickham, 2016).

### 2.5 Machine learning classification of Elongate dendritic

To further evaluate how phytolith traits relate to taxonomy, we tested whether Elongate dendritic morphometric traits could classify taxa at three hierarchical levels: (1) genus (lineage; see further), (2) species, and (3) subspecies within *Triticum* (**Table 1**), using Random Forest models (Breiman, 2001). Random Forest was chosen because it performs well with relatively small sample sizes and complex, correlated predictors (Qi, 2012). All analyses were conducted in R (R Core Team, 2024), with scripts archived on Zenodo.

**Table 1:** Class labels, sample distribution, and the morphometric traits used for Random Forest classification at three taxonomic levels: Lineage, Species, and *Triticum* subspecies.

Classification	Class labels with counts (train|validation|test|test_other)	Trait sets used for Random Forest
Lineage (6 classes) • Train entries: 525 • Validation entries: 158 • Test entries: 161 • Test_other entries: 287	*Avena* (180|59|55|0), *Hordeum* (129|42|40|0), *Secale* (187|60|59|0), *Triticum* (204|66|65|287), Poeae confusers (58|19|17|0), Triticodae confusers (196|64|63|0).	**Geometric trait set**= Volume, ConvexHullVolume, SurfaceArea, Solidity, Sphericity, MaxLength, MaxWidth, MaxHeight, PC1sd, PCS2sd, PC3sd, Elongation, Flatness, CoreVolume, CoreConvexVolume, CoreSurfaceArea, CoreOccupancy, CoreSolidity, CoreSphericity, CoremaxLength, CoreMaxWidth, CoreMaxHeight, CorePC1sd, CorePC2sd, CorePC3sd, CoreElongation, CoreFlatness. **Branching trait set**= AverageDendriticLength, DendriticDensity, DendriticNumber, DendriticTotalLength, **CC trait set** = CC_1, CC_2, CC_3, CC_4, CC_5, CC_6, CC_7, CC_8, CC_9, CC_10, CC_11, CC_12, CC_13, CC_14, CC_15, CC_16, CC_17, CC_18, CC_19, CC_20.
Species (39 classes) • Train entries: 477 • Validation entries: 175 • Test entries: 156 • Test_other entries: 991	*Aegilops biuncialis* (9|4|8|0), *A. cylindrica* (14|3|3|0), *A. geniculata* (13|5|2|0), *A. neglecta* (17|1|2|0), *A.* sp*eltoides* (9|5|6|20), *A. tauschii* (13|6|1|20), *Avena barbata* (16|5|2|22), *A. fatua* (15|8|1|40), *A. nuda* (13|5|2|0), *A. sativa* (11|4|5|41), *A. sterilis* (9|7|7|20), *A. strigosa* (15|4|1|20), *A. wiestii* (13|4|4|0), *Bromus erectus* (13|3|5|0), *B. ramosus* (12|3|7|0), *Dasypyrum villosum* (13|3|4|0), *Elymus caninus* (15|1|4|0), *E. repens* (12|3|5|0), *Helictochloa pratensis* (11|4|5|0), *Helictotrichon sedenense* (12|6|3|0), *Hordelymus europaeus* (16|3|1|0), *Hordeum marinum* (10|5|5|0), *H. murinum* (10|6|4|20), *H. secalinum* (12|6|2|0), *H. vulgare* (10|6|5|110), *Parapholis incurva* (11|4|5|0), *Secale africanum* (10|5|5|0), *S. anatolicum* (13|6|3|20), *S. cereale* (12|6|3|105), *S. segetale* (10|8|2|0), *S. strictum* (12|3|5|0), *S. sylvestre* (10|4|6|20), *S. vavilovii* (7|4|10|17), *Thinopyrum junceum* (14|5|2|0), *Triticum aestivum* (12|5|5|208), *T. monococcum* (13|4|3|61), *T. timopheevii* (15|1|4|20), *T. turgidum* (14|5|5|207), *T. urartu* (11|5|4|20).	
Subspecies *Triticum* (12 classes) • Train entries: 150 • Validation entries: 51 • Test entries: 47 • Test_other entries: 170	*Triticum aestivum* ssp. *compactum* (9|5|7|0), *T. aestivum* ssp. *macha* (14|1|5|0), *T. aestivum* ssp. sp*elta* (10|7|5|20), *T. aestivum* ssp. sp*haerococcum* (12|5|3|23), *T. monococcum* ssp. *aegilopoides* (14|5|1|20), *T. monococcum* ssp. *monococcum* (12|5|3|21), *T. timopheevii* ssp. *timopheevii* (12|2|6|20), *T. turgidum* ssp. *carthlicum* (13|4|3|0), *T. turgidum* ssp. *dicoccoides* (14|5|5|25), *T. turgidum* ssp. *dicoccum* (10|5|5|21), *T. turgidum* ssp. *durum* (15|4|2|20), *T. turgidum* ssp. *polonicum* (15|3|2|0).	

For each level, the number of classes and the number of samples in the training, validation, and test sets are reported, followed by the class labels with their respective sample counts in the format (train|validation|test|test_other).

The dataset was unevenly represented, with some groups contributing far more phytoliths than others (e.g., *Triticum* taxa alone accounted for 622 of the 1,850 phytoliths). In machine learning, such class imbalance is problematic because models tend to overfit larger groups while underrepresenting smaller ones, leading to biased predictions (Japkowicz and Stephen, 2002). To reduce this imbalance, we trimmed the majority groups to better match the smaller ones and removed groups with fewer than 20 phytoliths. For genus-level classification, we also merged some of the ‘potential confuser’ genera into two broader groups: those belonging to the subtribe Poeae (*Alopecurus*, *Arrhenatherum*, *Helictochloa*, *Helictotrichon*, *Lolium*, *Parapholis*) and those belonging to the supertribe Triticodae (*Aegilops*, *Bromus*, *Dasypyrum*, *Elymus*, *Hordelymus*, *Thinopyrum*), resulting in six classes: *Avena*, *Hordeum*, *Secale*, *Triticum*, Poeae, and Triticodae (**Supplementary Data Sheet 2**). Because this six-class system combines some genera into broader phylogenetic groupings, we hereafter refer to it as lineage-level classification.

After these steps, 1,563 phytoliths remained at the lineage level, 808 at the species level, and 248 at the *Triticum* subspecies level. Each dataset was partitioned into training (60%), validation (20%), and test (20%) sets (**Supplementary Data File 3**) using stratified sampling at the inflorescence sample level. Approximately 20 phytoliths per inflorescence sample were distributed across the three sets in an 60/20/20 ratio.

The trimmed phytoliths from the majority groups were retained as a second evaluation set, termed ‘test_other’ (**Supplementary Data Sheet 2**). The taxa represented in this set were the same as those used for training and validation, but the phytoliths themselves came from different plant specimens. The ‘test_other’ set therefore provided a stricter assessment of model generalization to phytoliths from new specimens of the same taxa.

Since both PH and CC traits quantify branching architecture—albeit in different ways—only one of the two sets was included as predictors alongside geometric predictors. Preliminary tests indicated that CC traits improved classification performance relative to PH traits. Although PH is theoretically more robust (Cohen-Steiner et al., 2007), CC was more computationally efficient and better suited to large datasets, while still demonstrating strong predictive power (Hacquard and Lebovici, 2024; Li et al., 2018). Models were implemented in the R package caret (Kuhn, 2008) and evaluated using classification accuracy and Cohen’s κ (Cohen, 1960). Confusion matrices were generated and visualized as heatmaps (Wickham, 2016).

**Figure 4:**
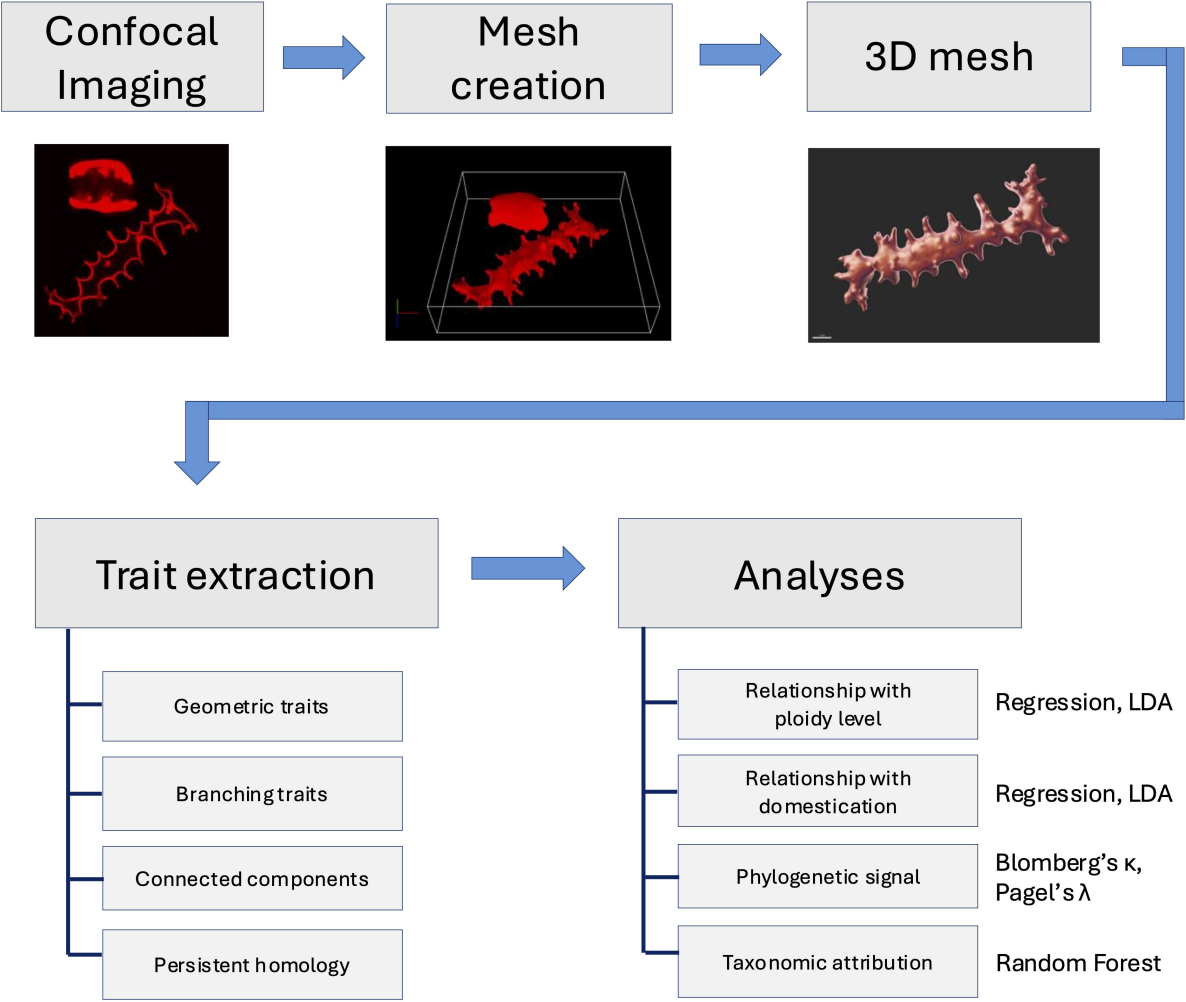
Schematic overview of the morphometric workflow used in this study.

A schematic overview of the morphometric workflow is presented in **Figure 4**, summarizing the main steps of the study, from sample selection and chemical processing through confocal imaging, 3D surface mesh generation, extraction of morphometric and topological traits, phylogenetic and regression analyses, and finally machine learning classification.

## 3 Results

### 3.1 Phylogenetic signal in Elongate dendritic

**Figure 5:**
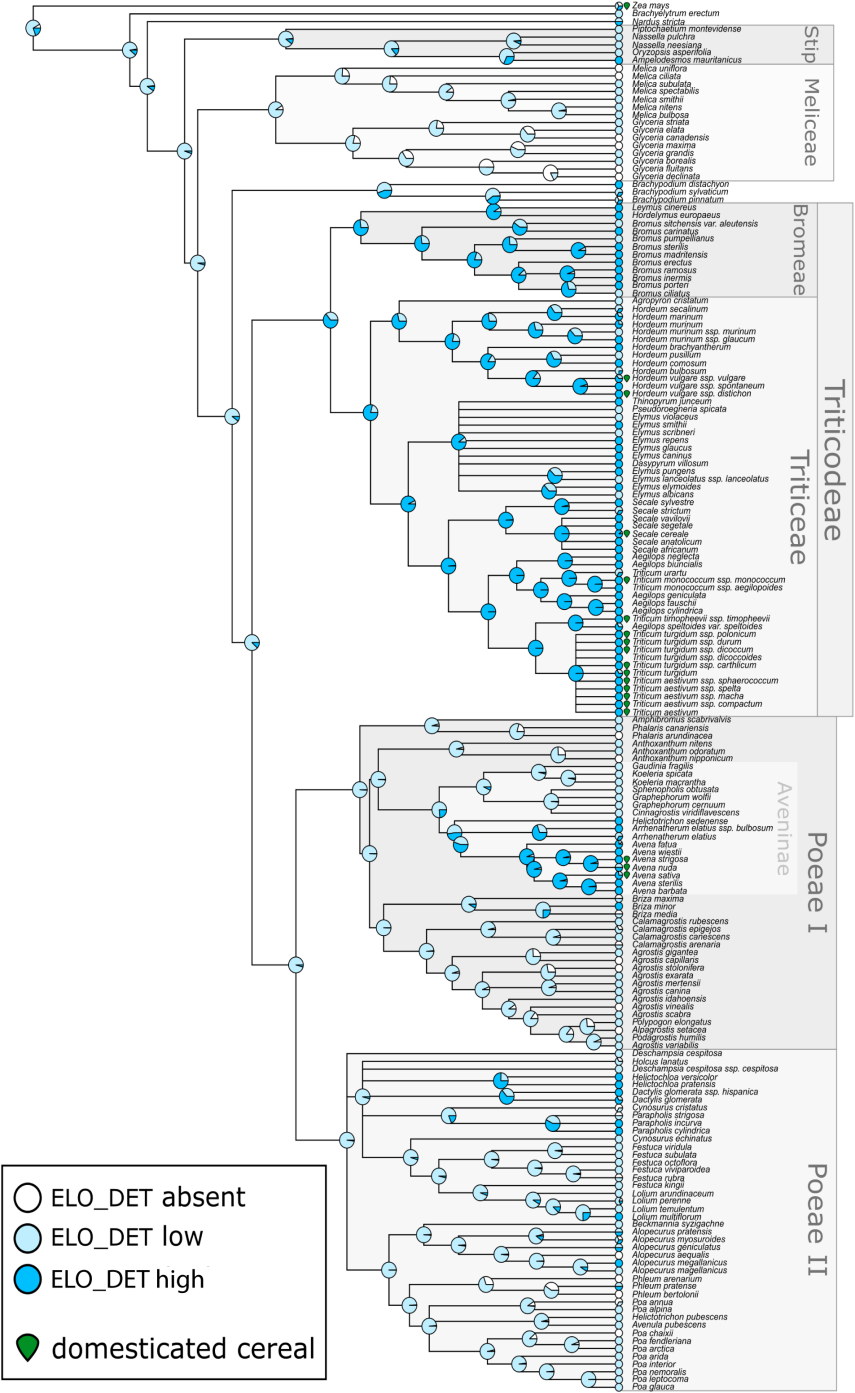
Molecular phylogeny of Pooideae grasses overlain with reconstructed ancestral states for level of Elongate dendritic production. Internal nodes show posterior probabilities of each production state from Bayesian threshold models, while tips show relative frequency of each type of production observed among sampled individuals from each species. The boundaries of major tribes (including the two subdivisions of tribe Poeae) are indicated along with the supertribe Triticodae and subtribe Aveninae. Species which are domesticated cereals are indicated by a green icon. Elo_det, Elongate dendritic.

Ancestral state reconstruction indicated that low occurrence of Elongate dendritic was likely ancestral within Pooideae (>85% posterior probability; **Figure 5**). Independent losses occurred in a few clades, such as Meliceae, while high production evolved multiple times independently, particularly within the Triticeae and Aveninae. The common ancestor of Triticeae likely had high Elongate dendritic occurrence (posterior probability = 0.79), as did the broader Triticodae crown group (posterior probability = 0.64). Similarly, Avena and several close relatives within Aveninae showed ancestrally high occurrence (posterior probability = 0.94). These results suggest that Elongate dendritic occurrence predates domestication, since it was already present in common ancestors, including the Triticodae crown group, which originated ~25 million years ago (Orton et al., 2021).

Out of 51 Elongate dendritic morphometric traits, 29 traits showed significant phylogenetic signal (λ = 0.98–0.46; mean = 0.75), indicating that closely related taxa tended to produce phytoliths with similar 3D morphometric traits. The strongest signals were observed in average dendritic length (λ = 0.98), solidity of the ‘core body’ (λ = 0.88), and maximum core length (λ = 0.86), while only four persistent homology (PH) traits (PH1, PH2, PH3, PH8) showed significant signal (λ = 0.85–0.68) (**Supplementary Data Sheet 3**).

**Figure 6:**
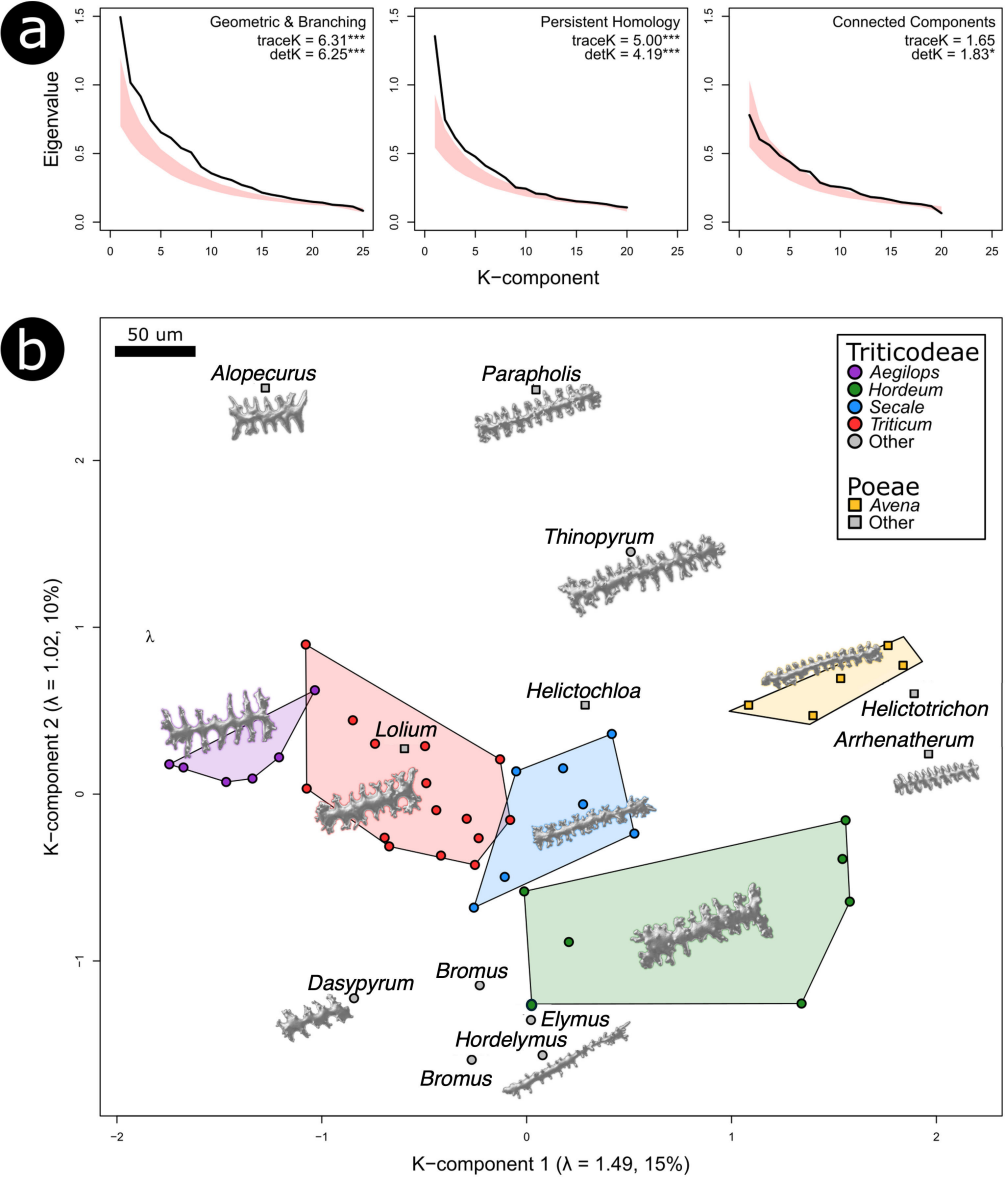
Multivariate analyses of phylogenetic signal. **(a)** Eigenvalues of the phylogenetic signal matrix K, calculated across three different partitions of the morphometric dataset, compared with 95% intervals of null distribution from permutation tests (shaded red). For each partition, the effect sizes for two summary statistics quantifying the degree of phylogenetic signal are provided, with indication if results were significantly different from the null of no phylogenetic structure (*p < 0.05, **p < 0.01, ***p < 0.001). Higher values indicate stronger phylogenetic structuring, with greater values of traceK relative to detK reflecting a more heterogeneous distribution of signal across morphometric traits **(b)** Distribution of species average morphometrics along the first two K-components calculated from the Persistent Homology dataset. For each plotted K-component, the eigenvalue (λ) and the % of phylogenetic signal it explains in are in the parentheses. Genera with at least one domestic cereal (including *Aegilops*) are colored, and examples of Elongate dendritic are given to scale in their approximate position within K-component morphospace.

The multivariate analyses showed that the geometric and branching traits carried the strongest phylogenetic signal overall, followed by the PH traits, while the connect components (CC) traits carried the weakest signal (**Figure 6a**). Both the geometric and branching set and the PH traits produced significant results for two summary statistics quantifying the degree of phylogenetic signal (traceK and detK), whereas the CC traits were significant only for detK. Taxa also separated clearly along the first two K-components, which represent trait combinations capturing the strongest phylogenetic structure (**Figure 6b**; Mitteroecker et al., 2025).

Within the geometric and branching trait set, surface area, volume, and solidity contributed most strongly to K1, while average dendritic length, number of dendritic branches, and surface area were most influential along K2. For the PH traits, PH19, PH9, and PH10 loaded highest on K1, whereas PH18, PH20, and PH12 dominated K2. The traits most strongly associated with the K-components did not always align with those highlighted by the univariate λ results, especially within the PH trait set. This likely reflects differences in how λ and κ capture phylogenetic signal, as well as the influence of trait variance in univariate analyses (Collyer et al., 2022). Despite these differences, both univariate and multivariate results consistently indicated that morphometric traits captured meaningful evolutionary relationships.

These results demonstrate that Elongate dendritic carry a clear phylogenetic imprint, both in their occurrence patterns and in their 3D morphometric features, with closely related taxa tending to resemble each other more strongly.

### 3.2 Domestication and morphometric variation

Domestication was associated with differences in Elongate dendritic morphometric traits. Binary logistic regression pointed to a link between domestication status (wild vs. domesticated) and morphometric traits. Seven rotated components (RCs), selected from a scree plot, were used as input factors (**Supplementary Data File 3**). The logistic regression model fit the data significantly better than the null model (χ²_7_ = 22.93, p = 0.0018). Four RCs predicted domestication status (p < 0.05; **Table 2**): RC4 increased the odds (OR = 3.37, 95% CI [1.18, 13.20]), while RC6 (OR = 0.58, 95% CI [0.34, 0.93]), RC2 (OR = 0.53, 95% CI [0.30, 0.87]), and RC7 (OR = 0.54, 95% CI [0.31, 0.89]) were associated with reduced odds. The remaining RCs (RC1, RC3, RC5) were not significant (**Table 2**).

**Table 2:** The first seven rotated components (RCs) (loaded with morphometric traits associated with domestication.

Predictor	B (log odds)	Standard error	Z	Odds ratio (95% CI)	p-value
Intercept	0.380	0.257	1.48	1.46 [0.90, 2.48]	0.138
RC3	–0.249	0.241	–1.03	0.78 [0.48, 1.24]	0.302
RC6	–0.551	0.249	–2.21	0.58 [0.34, 0.93]	0.027*
RC1	0.140	0.245	0.57	1.15 [0.71, 1.88]	0.569
RC4	1.214	0.606	2.00	3.37 [1.18, 13.20]	0.045*
RC2	–0.645	0.272	–2.37	0.53 [0.30, 0.87]	0.018*
RC5	–0.170	0.238	–0.72	0.84 [0.52, 1.34]	0.475
RC7	–0.611	0.266	–2.30	0.54 [0.31, 0.89]	0.021*

“Domesticated” is the reference category. *p <0.05.; CI, confidence interval.

**Figure 7:**
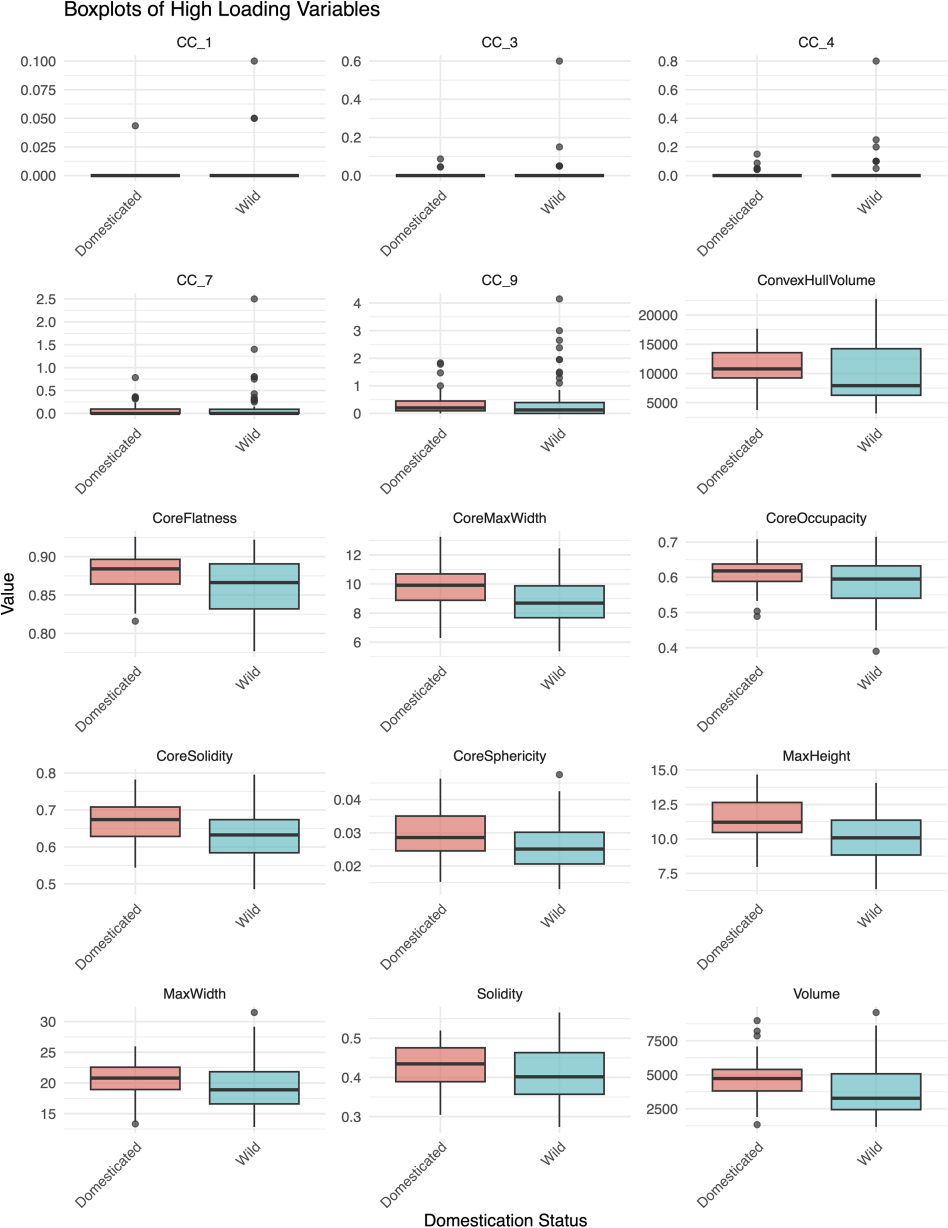
Boxplots of traits with high loadings (<0.60) on the significant rotated components (RCs) from the binary logistic regression model distinguishing domesticated and wild species.

This model suggested that the morphometric variation captured by specific rotated components (RC4, RC2, RC6, and RC7) may help discriminate between domesticated and wild species. RC2, highly loaded (loading > 0.60) by solidity of the ‘core body’, solidity, sphericity of the ‘core body’, and occupancy of the ‘core body’, indicated that domesticated taxa had a denser overall structure. RC7, highly loaded by flatness of the ‘core body’, indicated that wild taxa had a flatter inner structure. RC6, highly loaded by maximum height, maximum width, and maximum width of the ‘core body’, indicated that domesticated taxa generally had larger phytoliths. RC4 values, highly loaded by several CC traits, were higher in wild taxa (especially Aegilops), reflecting more complex dendritic branching in some wild taxa (**Figure 7**; **Supplementary Table S3**). However, some domesticated taxa, particularly within *Triticum*, also exhibited high CC values, suggesting that certain cultivated taxa retained extensive branching traits.

**Figure 8:**
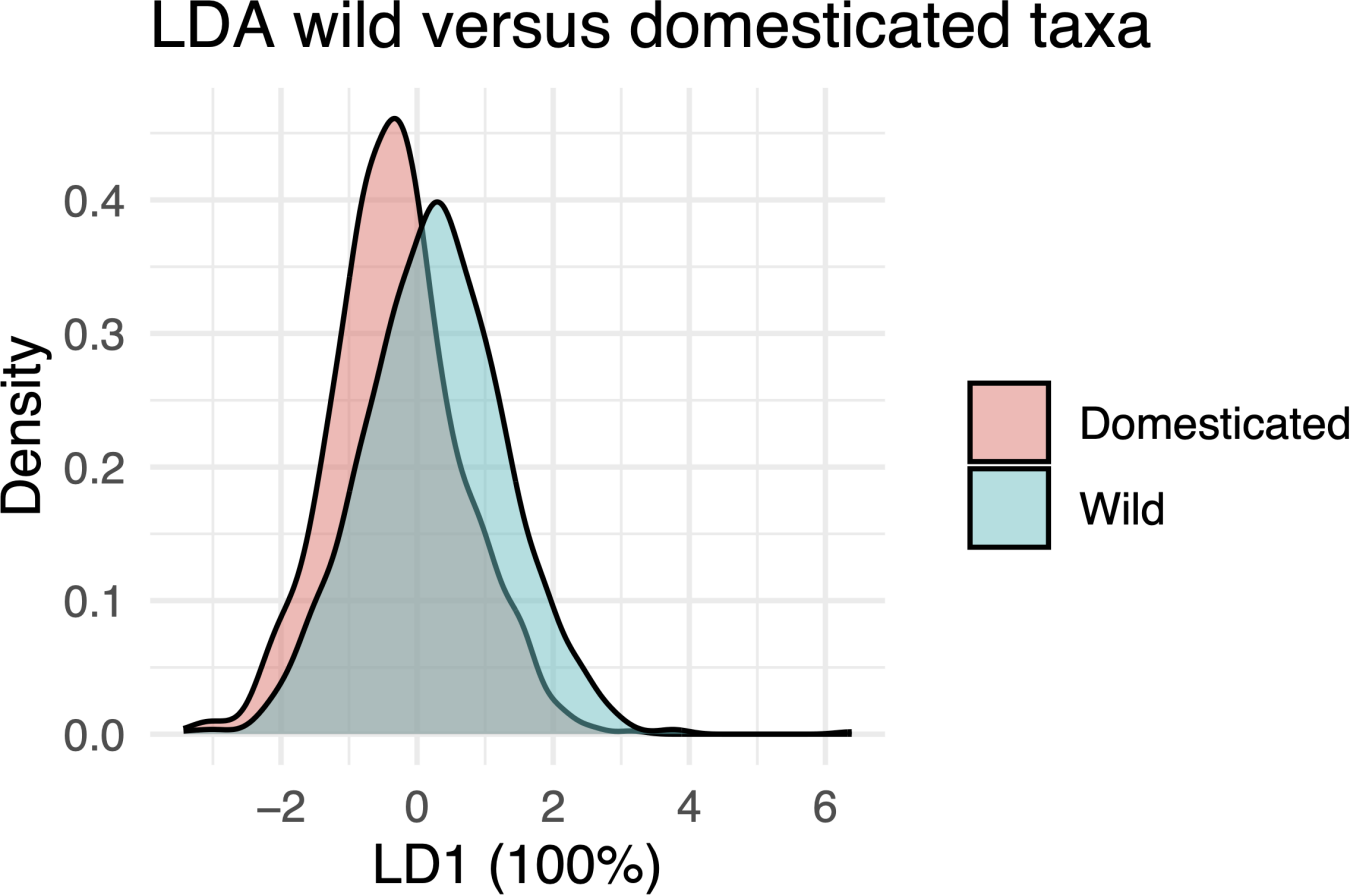
Linear Discriminant Analysis (LDA) plot showing density distributions along the first linear discriminant (LD1), which accounts for 100% of the discriminative power between wild and domesticated species.

Our LDA for domestication showed that LD1 provided some separation between wild and domesticated taxa. However, substantial overlap between individual phytoliths remained (**Figure 8**), suggesting that although domestication influenced morphometric traits, high within-group variation overlapping with between-group variation limited the ability to reliably distinguish individual phytoliths. The model achieved a classification accuracy of 62.3%, which was statistically higher than the no-information rate of 54% (binomial test, p < 0.001), but the improvement was modest.

### 3.3 Polyploidization and morphometric variation

To investigate the relationship between morphometric traits and ploidy level (diploid < tetraploid < hexaploid), we applied ordinal logistic regression. Seven RCs, selected from the scree plot, were used as predictors in a cumulative logit model (**Supplementary Table S3**). The ordinal logistic regression model did not significantly improve over the null model (χ²(7) = 5.30, p = 0.624). None of the RCs demonstrated a statistically significant effect, as their 95% confidence intervals all included 1 (the null hypothesis value) (**Table 3**). For example, RC1 had an odds ratio of 1.27 (95% CI [0.84, 1.95]), indicating that increases in RC1 did not significantly alter the odds of transitioning from one ploidy category to a higher one. Although the threshold between tetraploid and hexaploid was substantially greater than zero (log odds = 1.167, SE = 0.260, t = 4.493), this shift was not explained by any of the retained RCs.

**Table 3:** Rotated components (RCs) associated with ordered ploidy level (diploid < tetraploid < hexaploid).

Parameter	B (log odds)	Standard error	t-value	Odds ratio (95% CI)
RC3	0.222	0.21	1.06	1.25 [0.83, 1.90]
RC4	–0.286	0.3	–0.95	0.75 [0.33, 1.21]
RC6	0.060	0.21	0.29	1.06 [0.71, 1.59]
RC1	0.239	0.21	1.12	1.27 [0.84, 1.95]
RC2	0.076	0.21	0.36	1.08 [0.72, 1.65]
RC5	0.142	0.2	0.70	1.15 [0.78, 1.74]
RC7	0.214	0.21	1.01	1.24 [0.82, 1.90]
diploid | tetraploid	–0.076	0.22	–0.34	–
tetraploid | hexaploid	1.167	0.26	4.49	–

The intercepts (“diploid | tetraploid” and “tetraploid | hexaploid”) represent the latent-scale thresholds for transitioning from one ploidy category to the next. CI, confidence interval.

**Figure 9:**
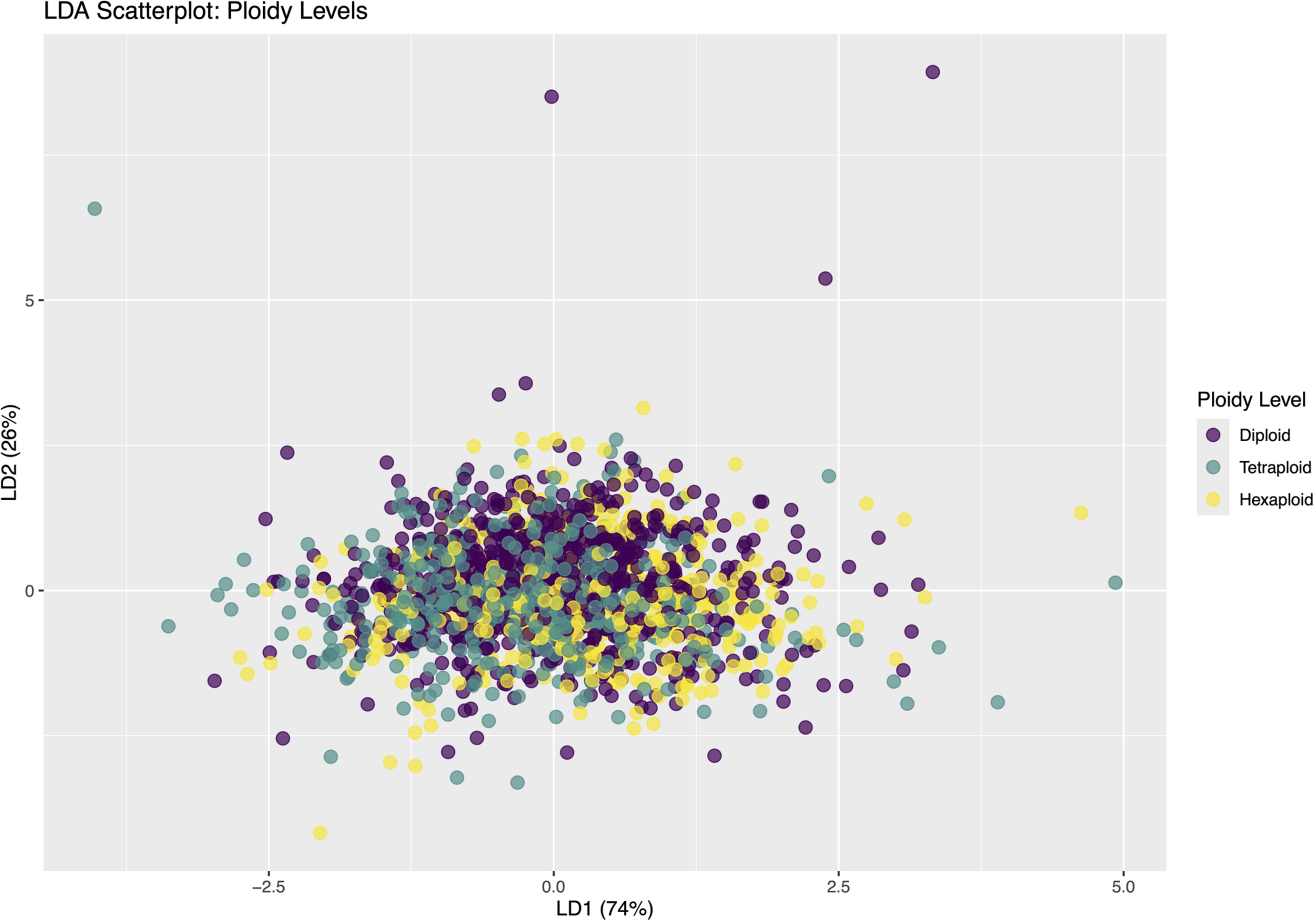
Linear Discriminant Analysis (LDA) scatterplot of observations colored by ploidy level. The first two linear discriminants, LD1 (74%) and LD2 (26%), capture the variance used to discriminate between diploid, tetraploid, and hexaploid groups.

The LDA for ploidy level revealed minimal separation among individual phytoliths from diploid, tetraploid, and hexaploid taxa, with substantial overlap between groups (**Figure 9**). A binomial test indicated that the observed accuracy (48.6%) was not significantly greater (p = 0.243) than the no-information rate (NIR = 47.7%), suggesting that ploidy level did not strongly influence the morphometric traits of individual Elongate dendritic.

### 3.4 Taxonomic attribution using morphometric traits

All Random Forest models performed significantly better than chance (p < 0.05 across all tests; **Table 4**). The best classification performance was achieved at the lineage level (six classes; 844 entries), where the model reached a test accuracy of 49.5%, a strong improvement over the no-information rate (21.7%). At the species level (39 classes; 808 entries), classification accuracy decreased to 34.0% but remained far above the no-information rate (6.4%). At the Triticum subspecies level, the test accuracy was 31.9%, compared to a no-information rate of 14.9%.

**Table 4:** Summary of the Random Forest classification performance across taxonomic levels (Lineage, Species, *Triticum* Subspecies).

Classification	Set	Accuracy	Kappa	NIR	p-value
Lineage	Validation	49.0%	0.37	21.3%	<0.001*
Lineage	Test	49.5%	0.38	21.7%	<0.001*
Species	Validation	32.0%	0.30	4.6%	<0.001*
Species	Test	34.0%	0.32	6.4%	<0.001*
Subspecies *Triticum*	Validation	37.3%	0.32	13.7%	<0.001*
Subspecies *Triticum*	Test	31.9%	0.25	14.9%	<0.001*

Accuracy and Kappa represent the overall classification performance. The No Information Rate (NIR) indicates the expected accuracy when always predicting the most frequent class. The P value tests whether model accuracy is significantly higher than the NIR (*p <0.05).

Following the evaluation framework of Landis and Koch (1977), the test kappa values for lineage (0.38), species (0.30), and *Triticum* subspecies (0.25) indicated a fair level of agreement (κ ranging from 0.21 to 0.40) between predicted and true classes. Although classification accuracy declined with increasing taxonomic resolution, morphometric traits retained taxonomic signal across all levels. The reduced accuracy likely reflects either limited taxonomic resolution of phytoliths at finer scales or insufficient training data at lower taxonomic levels, where many classes had few samples.

The confusion matrices showed that, at the lineage level, *Avena* was sometimes misclassified as *Secale*, followed by Triticodae confusers, *Hordeum*, and *Triticum*. Poeae confusers, by contrast, were well separated. *Hordeum* was primarily misclassified as *Avena* or Triticodae confusers. *Secale* was commonly misclassified as *Triticum* or Triticodae confusers. *Triticum* was misclassified mainly as Triticodae confusers, but also as *Secale* and, to a lesser extent, as *Hordeum* or *Avena*. Finally, Triticodae confusers were most frequently misclassified as *Triticum*, but also as *Hordeum* and *Secale* (**Supplementary Table 4**). At the species and *Triticum* subspecies levels, misclassifications were more scattered, likely reflecting greater trait overlap across taxa and the increased classification complexity due to the higher number of classes (**Supplementary Table S4**).

**Figure 10:**
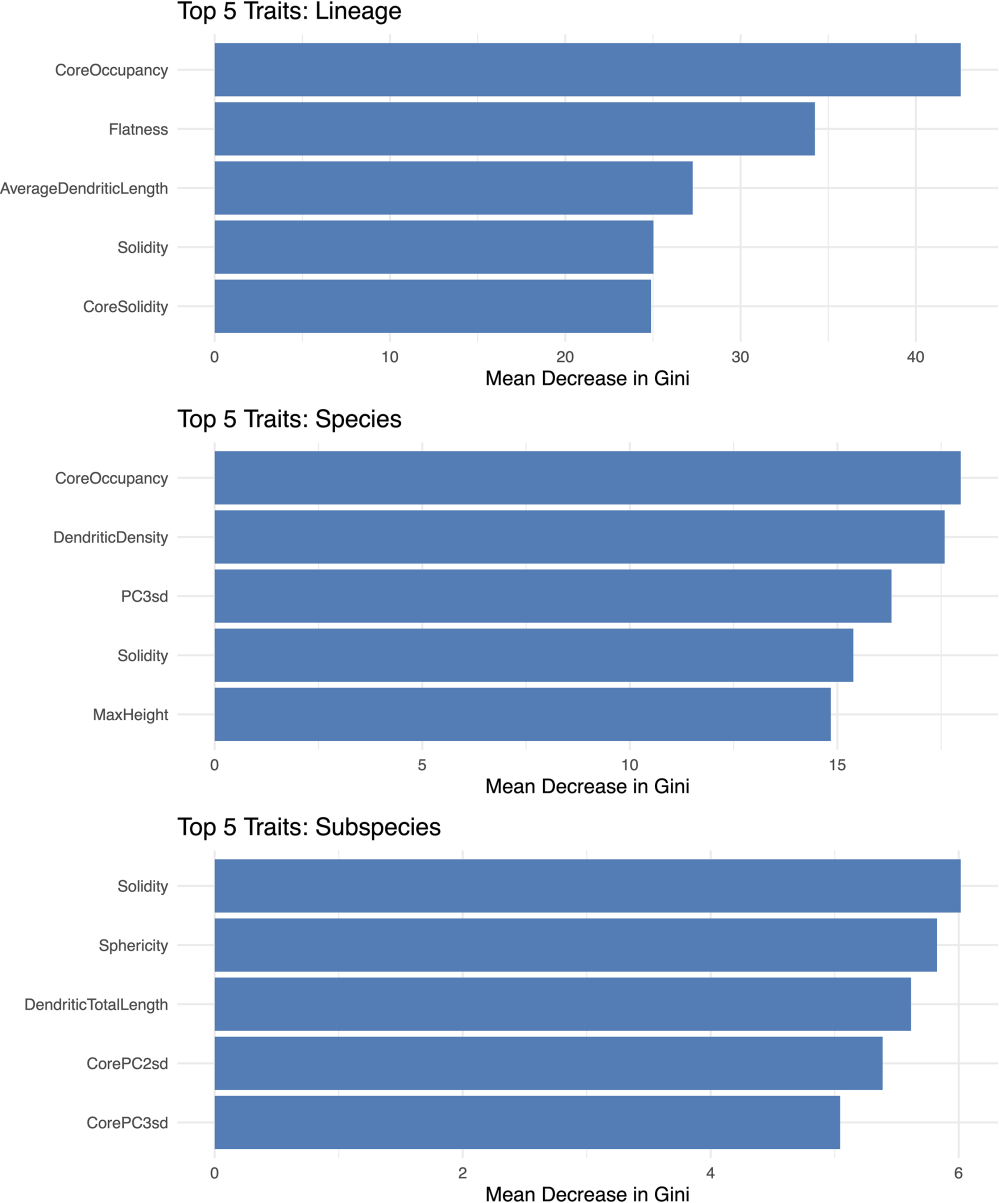
Trait importance contributing to phytolith classification at the lineage, species, and *Triticum* subspecies levels, based on Random Forest models trained on geometric and topological traits. Trait importance is expressed as the mean decrease in Gini impurity, reflecting each trait’s relative contribution to improving classification performance by reducing class uncertainty at each decision split.

To identify which traits the models relied on most, we examined the top five importance scores for the classification of lineages, species, and *Triticum* subspecies (**Figure 10**). At the lineage level, the most important traits were ‘core body’ occupancy (relative to the entire phytolith), flatness, average dendritic branch length, and the solidity of both the whole phytolith and the ‘core body’. At the species level, the top-ranked traits included occupancy of the ‘core body’, dendritic branch density, and the standard deviations along the second and third principal components—related to phytolith width and height variation, respectively. For Triticum subspecies, the most influential traits were solidity, sphericity, total dendritic length, and the standard deviations along the second (~width) and third (~height) principal components of the ‘core body’.

To evaluate model generalizability, we tested classification accuracy on phytoliths from completely unseen inflorescence samples—i.e., those held out during the initial split due to being majority groups. While these particular inflorescence samples were not seen during training, they still belonged to taxa represented in the training set. At the lineage level, 287 *Triticum* phytoliths were included in the test_other set. The model correctly classified 53% of them as *Triticum*, while the remainder were misclassified as Triticodae confusers and, to a lesser extent, as *Secale*, *Avena*, or *Hordeum* (**Supplementary Table S4**).

At the species level, we evaluated 991 phytoliths from 18 unseen species. Accuracy on this test_other set was 9.7%, considerably lower than the 33.9% accuracy observed on the standard test set. The observed accuracy did not exceed the no-information rate of 21.0% (p = 1.00), indicating that the model failed to generalize to these unseen taxa. This was likely the result of limited training data, where only a single inflorescence sample per species was included. Our results suggest that comparing phytoliths from an unseen specimen of a given species to just one specimen in the training set is insufficient for robust prediction. For *Triticum* subspecies, the test_other set included phytoliths from eight previously unseen samples. Accuracy was 15.8%, which did not differ significantly from the no-information rate (14.7%; p = 0.36), indicating that the model performed only marginally better than chance on these unseen individuals. As with the species-level model, the limited training data (only one inflorescence sample per subspecies) likely restricted model performance (**Supplementary Table S4**).

## 4 Discussion

### 4.1 Occurrence of Elongate dendritic

In this study, we evaluated the distribution of Elongate dendritic across Pooideae inflorescence samples. The occurrence categories applied here (absent, low, high) should be regarded as estimates, with the primary aim of identifying where Elongate dendritic occurred across the Pooideae to ensure representative sampling for morphometric analysis. Nonetheless, these estimates revealed that high occurrence of Elongate dendritic appears to have evolved independently in multiple cereal-rich clades, including Triticeae and *Avena*. In contrast, complete absence was rare, observed only in a few lineages such as Meliceae, while many lineages exhibited low occurrence. This evolutionary pattern illustrates that the capacity to produce Elongate dendritic was phylogenetically widespread across Pooideae, but high occurrence was concentrated in select lineages—many of which include important domesticated cereals such as wheat, barley, rye, and oats, as well as non-cereal taxa (**Figure 5**). These findings align with Novello and Barboni (2015), who reported that Elongate dendritic occurred frequently in wild African grasses. In archaeological contexts, Elongate dendritic should therefore not be automatically interpreted as evidence for cereals. This perspective is consistent with ICPN 2.0 guidelines, which recommend cereal attribution only in secure contexts, such as storage or cooking vessels, or specific thin sections (ICPT, 2019).

Albert et al. (2008) reported that an Elongate dendritic relative abundance of around 7–8% within a phytolith assemblage may indicate domesticated cereals. In future work, we aim to revisit the inflorescence samples and quantify relative abundances of Elongate dendritic more precisely. This will allow us to test whether relative abundances are consistently linked to domestication status or if they reflect deeper phylogenetic structuring.

### 4.2 Morphometric traits and their diagnostic value

Our results supported the first part of hypothesis H1, that domestication influences the morphometric traits of Elongate dendritic. Domesticated taxa generally produced larger, denser, and flatter phytoliths, while some wild taxa exhibited more complex dendritic branching (**Figure 7**). Nonetheless, LDA revealed substantial morphometric overlap at the individual level, highlighting high intra-sample variation and the limited reliability of single phytoliths for distinguishing wild from domesticated forms (**Figure 8**). These results are consistent with earlier studies linking domestication to increased phytolith size (Piperno, 1988; Piperno and Stothert, 2003; Pearsall, 1989; Vrydaghs et al., 2009), although more recent work on rice has shown considerable size overlap between wild and domesticated populations (Wang et al., 2019).

Our results did not support the second part of hypothesis H1, that ploidy level influences the morphometric traits of Elongate dendritic. No consistent morphometric patterns were detected across ploidy levels, suggesting that polyploidy had little effect on Elongate dendritic traits (**Table 3**; **Figure 9**). As ploidy information was obtained from the literature, future work should include cytogenetic verification to refine these findings (Diet et al., 2022).

Our results partially supported hypothesis H2, that 3D morphometric traits of Elongate dendritic can distinguish taxa at different taxonomic levels within subfamily Pooideae. We detected significant phylogenetic signal in several traits, including solidity, core body solidity, maximum core body length, volume, surface area, average dendritic length, and number of dendritic branches (**Figure 6**; **Supplementary Data Sheet 2**). Many of these traits also emerged as important for classification across taxonomic groups (**Figure 10**). Among the classification approaches tested, Random Forest models performed best at the lineage level (accuracy 49.5%, κ = 0.38), with performance decreasing at the species (accuracy 34.0%, κ = 0.32) and *Triticum* subspecies levels (accuracy 31.9%, κ = 0.25) (**Table 4**). These results support earlier findings that phytolith morphometry contains phylogenetically informative variation (Brightly et al., 2024; Chen et al., 2020; Gallaher et al., 2020; Hošková et al., 2020, 2021; Vrydaghs et al., 2009). The strong phylogenetic structuring of Elongate dendritic morphometric traits may also explain why it was difficult to separate wild from domesticated taxa, given their close taxonomic relatedness.

### 4.3 Towards improved taxonomic attribution of Elongate dendritic

The results of this study are promising, indicating that classification based on isolated Elongate dendritic is feasible, particularly at the lineage level. However, the accuracy values achieved by our models were not necessarily high enough to be reliable for archaeological inference (**Table 4**).

A likely explanation for the moderate classification accuracy, particularly at the species and subspecies levels, is the limited number of phytoliths analyzed per sample (approximately 20 per inflorescence). Such small sample sizes may not adequately capture the considerable intra-specimen morphometric variation. Increasing the number of phytoliths per specimen would likely mitigate this limitation. However, the use of 3D confocal microscopy remains both time-intensive and resource-demanding, which constrains the scalability of this approach. As a practical alternative, a 2D framework based on standard light microscopy could be developed, incorporating either manual or automated outlining of individual Elongate dendritic (see, e.g., Lloyd et al., 2024), followed by geometric and topological data analysis. Although this approach sacrifices three-dimensional detail, it would enable the acquisition of substantially larger and more representative datasets. Moreover, it would differ from earlier 2D analyses by Ball et al. (2017), which examined dendritic lobes in articulated phytoliths, by focusing instead on isolated Elongate dendritic, which broadens the number and type of samples that can be analyzed.

Future work could explore deep learning approaches which learn directly from 3D meshes such as MeshCNN (Hanocka et al., 2019), rather than relying on predefined features. Deep learning methods have shown promise for Elongate dendritic in 2D, both for morphotype recognition of isolated phytoliths (Andriopoulou et al., 2023) and taxonomic attribution of silica skeletons containing Elongate dendritic (Berganzo-Besga et al., 2022). However, applying deep learning approaches to our 3D dataset may also present challenges, including overfitting due to small sample sizes, preprocessing demands (e.g., mesh standardization), high computational requirements, and the black-box nature of deep learning algorithms in general.

Regarding model evaluation, the ‘test_other’ set—composed of phytoliths from the same taxa as those used for training but collected from different individual plants—provides a useful check on how well the model generalizes to new specimens of familiar taxa (**Supplementary Table S4**). Currently, it represents only a subset of the taxa used for training and does not include any entirely new taxa outside the training set, so it cannot evaluate performance on species outside the training set. Expanding this set to include all taxa present in the training set, as well as taxa not used for training, would allow a more comprehensive assessment of model performance across both familiar and entirely new taxa. It is also important to evaluate how the model perform on Elongate dendritic originating from different biological, environmental or depositional contexts. These may influence morphometric traits in ways that affect classification performance. Several of these sources of variation are discussed below.

### 4.4 Sources of morphometric variation in Elongate dendritic

A range of factors may influence morphometric variation in Elongate dendritic, many of which we could not control in this study. For example, previous work has shown that dendritic morphometric traits vary within the inflorescence across bract types (glumes, lemmas, paleae), across spikelet or panicle positions (upper, middle, lower), and even between accessions of the same species (Ball et al., 2017). Other factors may influence Elongate dendritic morphology but are largely unstudied.

First, environmental conditions are known to influence phytolith morphology, with size often varying in response to factors such as soil moisture, temperature, and light regime, while shape appears comparatively stable across these gradients (Ball and Brotherson, 1992; Dunn et al., 2015; Hošková et al., 2021; Liu et al., 2016). For Elongate dendritic, however, no studies have specifically addressed how environmental or biogeochemical factors affect their morphometric traits. In addition, factors such as silica availability, nutrient balance, and lignification may be important, but remain to be investigated for Elongate dendritic.

Second, plant developmental stage may contribute to variation. For example, bulliform phytoliths in rice vary in size and ornamentation across growth stages (He et al., 2024), although Hošková et al. (2020) found minimal ontogenetic variation in Bilobate and Polylobate forms. Controlled growth experiments across developmental stages and inflorescence bract types (glumes, lemmas, paleae) will be essential to clarify how such variation might affect taxonomically informative traits.

Third, post-depositional and laboratory-induced alterations warrant consideration. Natural and anthropogenic post-depositional processes, including physical mechanisms such as breakage and erosion, chemical effects such as dissolution, and exposure to heat, could alter the morphology of Elongate dendritic (Cabanes et al., 2011; Cabanes and Shahack-Gross, 2015; Madella and Lancelotti, 2012; Wang and Shang, 2023). These alterations may affect features such as the dendritic branches; the psilate or nodulate surface texture; and the overall length of the phytolith, which may be shorter due to breakage. Laboratory preparation methods may also introduce morphological changes. Some samples were processed using wet oxidation alone, while others underwent a combination of wet and dry oxidation. Dry oxidation may cause deformation (shrinkage) in some phytoliths (Pearsall, 1989), likely due to dehydration during heating, although Parr et al. (2001) found no difference in Bilobate size between dry and wet oxidation. In addition, in modern inflorescence samples, isolated Elongate dendritic exhibit broken or missing dendritic processes or can be fractured in length, meaning their morphology is not always perfectly intact, even in reference material. Understanding how these processes affect the morphometric traits of Elongate dendritic, and whether such changes influence taxonomic attribution by classification models, is crucial.

Finally, broader taxonomic comparisons could help contextualize observed variation. While Elongate dendritic are most commonly produced in the inflorescence bracts of Pooideae, they have also been documented in other Poaceae subfamilies and, more sporadically, in unrelated monocot families such as Marantaceae and Arecaceae (Novello and Barboni, 2015; Ge et al., 2020; ICPT, 2019). Light microscopy could provide a first assessment of whether these forms are morphologically comparable to those in Pooideae. Regardless, biogeographic and ecological context remains essential: for example, Elongate dendritic found in tropical families such as Marantaceae are unlikely to be relevant in temperate archaeological contexts, where such plants would not have been present. Likewise, Elongate dendritic found in phytolith assemblages lacking Spheroid echinate or Spheroid ornate but containing grass silica short cells, are most likely derived from grasses.

### 4.5 Implications for archaeological applications

As the Elongate dendritic dataset continues to grow and morphometric variation becomes better characterized, the next step should be to apply classification models to archaeological assemblages. Crucially, this does not require the inclusion of all species analyzed in this study. Our broad sampling strategy, designed to test generalizability across Pooideae, inevitably introduced high morphometric variation and thereby lowered classification accuracy. In contrast, archaeological contexts typically involve a narrower set of expected taxa, defined by region, chronology, and other contextual evidence. This restricted scope opens the possibility of building focused, high-accuracy models tailored to specific case studies. For example, in a medieval Northwest European context, there would be no need to include non-local progenitor taxa such as *Triticum urartu* or *T. turgidum* subsp. *carthlicum*, and excluding non-applicable taxa could significantly improve model performance.

More broadly, we hope that future archaeological research can integrate cereal taxonomic questions more explicitly into phytolith analysis. An ideal future outcome from this project would be the development of a tool with a user-friendly interface for attributing isolated Elongate dendritic, one that reduces reliance on extensive human training or subjective visual comparison. By leveraging models trained on morphometric patterns, such tools could standardize identifications, reduce inter-observer bias, and make phytolith analysis more accessible to a wider range of researchers. In turn, this would support more consistent and reproducible interpretations in archaeological contexts.

## 5 Conclusion

This study provided the first large-scale quantitative assessment of Elongate dendritic across Pooideae, integrating 3D morphometrics, topological descriptors, phylogenetic reconstruction, and machine learning. Our results indicated that low Elongate dendritic occurrence was likely the ancestral trait within Pooideae, with high occurrence levels evolving independently in multiple cereal-rich lineages, including Triticeae and Aveninae.

We tested the potential of 3D morphometric and topological analysis of Elongate dendritic for taxonomic attribution. Lineage-level classification achieved moderate accuracy, but high intra-sample variation and morphometric overlap between taxa limited performance at finer taxonomic resolutions (species and *Triticum* subspecies). Domestication appeared to be associated with shifts in average phytolith size and shape, but no consistent morphometric trends were linked to polyploidy.

Future work should include growth experiments to disentangle sources of morphometric variation and to improve classification accuracy by increasing sample sizes and better capturing intra-sample variability. Given the time and cost of 3D imaging, an equivalent 2D approach that incorporates topological analysis could provide a more scalable and accessible alternative. Overall, these results contributed to investigating the taxonomic utility of Elongate dendritic and to advancing their application in both evolutionary and archaeological research.

## Data Availability

The datasets presented in this study can be found in online repositories. The names of the repository/repositories and accession number(s) can be found in the article/**Supplementary Material**. The confocal images, 3D surface meshes, R scripts, and input data used in this study are available on Zenodo: Confocal images repository 1: https://doi.org/10.5281/zenodo.13870271. Confocal images repository 2: https://doi.org/10.5281/zenodo.13920175. 3D surface meshes, R scripts and input data for the scripts: https://doi.org/10.5281/zenodo.15620090. The custom MATLAB scripts used for the trait extraction can be found at https://github.com/maoli0923/Phytolith_3D. The 3D surface meshes can be found at MorphoSource (project ID: 000622427), which offers a user-friendly interface. https://www.morphosource.org/projects/000622427?locale=en.

## Supplementary material

Supplementary Table 1 Sample table including taxon name, sample number, Genbank name and data, domestication labels, ploidy level labels, collection information, taxonomic information, Elongate dendritic occurrence, number of 3D images per sample, and phytolith extraction protocol used.

Supplementary Table 2 Overview and descriptions of morphometric traits.

Supplementary Table 3 Morphometric trait reduction, component selection, and model evaluation for domestication and ploidy models.

Supplementary Table 4 Confusion matrices for lineage-, species-, and Triticum subspecies-level classifications for the test and test_other set, and output metrics for test_other set.

Supplementary Data Sheet 1 Model comparison of molecular clock analyses.

Supplementary Data Sheet 2 Overview of the morphometric dataset. Each row represents morphometric traits from an individual 3D phytolith model.

Supplementary Data Sheet 3 Pagel’s λ estimates for phylogenetic signal across 51 morphometric traits (geometric, branching, and PH traits).
