# Supplementary material for: Elongate dendritic phytoliths as indicators for cereal identification and domestication: exploring a 3D morphometric approach
Source: Front Plant Sci. 2025 Oct 6;16:1643447. doi: 10.3389/fpls.2025.1643447 (PMC12535970; doi:10.3389/fpls.2025.1643447)
Supplement: Supplementary Table 2 — Overview and descriptions of morphometric traits. [file Table2.docx]

**SI Table 2: Overview and descriptions of morphometric traits. PH = Persistent Homology; CC = Connected Components.**

| **Trait** | **Unit** | **Type** | **Type of feature** | **Description and calculation** |
| --- | --- | --- | --- | --- |
| Volume | µm^3^ | size | geometric | This is the three-dimensional space that the phytolith (triangular mesh) holds. It is the estimation of the total voxels derived from the surface mesh multiplied by the unit³ (µm) of each voxel side. |
| ConvexHullVolume | µm^3^ | size | geometric | The convex hull is the smallest convex shape that completely encloses the phytolith. It is calculated from the volume of the smallest convex set that contains all the voxels comprising the phytolith. |
| SurfaceArea | µm^2^ | size | geometric | This is the total area covered by the outer surface of the phytolith. It is the sum of the areas of the triangle faces on the surface mesh. |
| Solidity | N.A. | shape | geometric | This is a measure of the compactness of the phytolith its shape. It is calculated as the ratio Volume/ConvexHullVolume. |
| Sphericity | N.A. | shape | geometric | This is a measure of how closely the shape of the phytolith approximates a perfect sphere. It is calculated by the formula 6π(Volume)^2^/(SurfaceArea)^3^. |
| MaxLength | µm | size | geometric | This is the maximum range along PC1 which is the maximum PC1 value minus the minimum PC1 value. |
| MaxWidth | µm | size | geometric | For phytoliths having an elongation<0.5, MaxWidth is the maximum range along PC2. For phytoliths having an elongation>0.5, the phytolith is partitioned into three regions along PC1. The maximum range along PC2 for every region is calculated. MaxWidth is the maximum value among those three ranges. |
| MaxHeight | µm | size | geometric | For phytoliths having an elongation<0.5, MaxHeight is the maximum range along PC3. For phytoliths having an elongation>0.5, the phytolith is partitioned into three regions along PC1. The maximum range along PC3 for every region is calculated. MaxHeight is the maximum value among those three ranges. |
| PC1sd | µm | size | geometric | Standard deviation along the first principal component, which captures the most significant source of variation in the 3D mesh of the phytolith. This axis often represents the largest trend or primary morphological feature of the structure, in most cases the length of the phytolith. |
| PC2sd | µm | size | geometric | Standard deviation along the second principal component, which captures the second most significant variation in the 3D mesh of the phytolith, typically orthogonal to PC1, in most cases the width of the phytolith. |
| PC3sd | µm | size | geometric | Standard deviation along the third principal component, which captures the third most significant source of variation in the 3D mesh of the phytolith, adding further detail to the shape’s structure, in most cases the height of the phytolith. |
| Elongation | N.A. | shape | geometric | Elongation compares the standard deviations along the second and first principal components. Since PC1 often represents the primary axis of variation (length), and PC2 captures the second most significant dimension (often width), this ratio provides insight into the relative spread of the shape in these two directions. This is calculated as ratio PC2sd / PC1sd. A lower value (closer to 0) would indicate a more elongated shape along PC1, whereas a value closer to 1 suggests a shape that’s more balanced or equidimensional in both directions. |
| Flatness | N.A. | shape | geometric | Flatness compares the standard deviations along the third and second principal components. Since PC2 often represents the secondary axis of variation (width), and PC3 captures the third significant dimension (height), this ratio provides insight into the relative spread of the shape in these two directions. This is calculated as the ratio PC3sd / PC2sd. A lower value (closer to 0) would indicate a flatter shape along PC2, whereas a value closer to 1 suggests a shape with more balanced thickness across both dimensions. |
| CoreVolume | µm^3^ | size | geometric | This is the three-dimensional space that the ‘core body’ of the phytolith holds. It is the estimation of the total voxels derived from the surface mesh of the ‘core body’ multiplied by the unit³ (µm) of each voxel side. |
| CoreConvexHullVolume | µm^3^ | size | geometric | The convex hull is the smallest convex shape that completely encloses all the voxels comprising the ‘core body’ of the phytolith. It is calculated from the volume of the smallest convex set that contains all the voxels comprising the ‘core body’. |
| CoreSurfaceArea | µm^2^ | size | geometric | This is the total area covered by the outer surface of the ‘core body’ of the phytolith. It is the sum of the areas of the triangle faces on the ‘core body’ surface mesh. |
| CoreOccupancy | N.A. | shape | geometric | This is a measure of the density or ‘occupancy’ of the ‘core body’ within the overall volume of the phytolith. It is calculated as the ratio CoreVolume/Volume. |
| CoreSolidity | N.A. | shape | geometric | This is a measure of the compactness of the ‘core body’ of the phytolith its shape. It is calculated as the ratio CoreVolume/CoreConvexHullVolume. |
| CoreSphericity | N.A. | shape | geometric | This is a measure of how closely the shape of the ‘core body’ of the phytolith approximates a perfect sphere. It is calculated by the formula 6π(Volume)^2^/(SurfaceArea)^3^. |
| CoreMaxLength | µm | size | geometric | This is the maximum range along PC1 of the ‘core body’ which is the maximum PC1 value minus the minimum PC1 value. |
| CoreMaxWidth | µm | size | geometric | For phytoliths having a CoreElongation<0.5, the ‘core body’ is partitioned into three regions along PC1. The maximum range along PC2 for every region is calculated. CoreMaxWidth is the maximum value among those three ranges. For phytoliths having a CoreElongation>0.5, the CoreMaxWidth is the maximum range along PC2. |
| CoreMaxHeight | µm | size | geometric | For phytoliths having a CoreElongation<0.5, the ‘core body’ is partitioned into three regions along PC1 and the maximum range along PC3 for every region is calculated. CoreMaxHeight is the maximum value among those three ranges. For phytoliths having a CoreElongation>0.5, the CoreMaxHeight is the maximum range along PC3. |
| CorePC1sd | µm | size | geometric | Standard deviation along the first principal component, which captures the most significant source of variation in the 3D mesh of the ‘core body’ of the phytolith. This axis often represents the largest trend or primary morphological feature of the structure, in most cases the length of the ‘core body’. |
| CorePC2sd | µm | size | geometric | Standard deviation along the second principal component, which captures the second most significant variation in the 3D mesh of the ‘core body’ of the phytolith, typically orthogonal to PC1, in most cases the width of the ‘core body’. |
| CorePC3sd | µm | size | geometric | Standard deviation along the third principal component, which captures the third most significant source of variation in the 3D mesh of the ‘core body’, adding further detail to the shape’s structure, in most cases the height of the ‘core body’. |
| CoreElongation | N.A. | shape | geometric | Elongation compares the standard deviations along the second and first core principal components. Since CorePC1 often represents the primary axis of variation (length), and CorePC2 captures the second most significant dimension (often width), this ratio provides insight into the relative spread of the shape in these two directions. This is calculated as ratio CorePC2sd / CorePC1sd. A lower value (closer to 0) would indicate a more elongated shape along PC1, whereas a value closer to 1 suggests a shape that’s more balanced or equidimensional in both directions. |
| CoreFlatness | N.A. | shape | geometric | Flatness compares the standard deviations along the third and second core principal components. Since CorePC2 often represents the secondary axis of variation (width), and CorePC3 captures the third significant dimension (height), this ratio provides insight into the relative spread of the shape in these two directions. This is calculated as the ratio CorePC3sd / CorePC2sd. A lower value (closer to 0) would indicate a flatter shape along CorePC2, whereas a value closer to 1 suggests a shape with more balanced thickness across both dimensions. |
| DendriticNumber | N.A. | branching | topology | This is the total number of bars that are longer than 1 µm in the persistence barcode. |
| DendriticDensity | N.A. | branching | topology | The dendritic density is calculated as the DendriticNumber/MaxLength providing a measure of branch complexity relative to the primary structural variation. |
| DendriticTotalLength | µm | branching | topology | The TotalBranchLength is the sum of the length of all branches that are longer than 1 µm. Larger value usually shows more branches or longer branches. We first computed the persistent barcode with geodesic distance to the ‘core body’ of the phytolith in which each bar represents a branch with its length information recorded. Then, we summed the length of all bars which are longer than 1 µm. |
| AverageDendriticLength | µm | branching | topology | Larger value of the average dendritic length usually means overall longer dendritic branches. It is calculated by the ratio DendriticTotalLength /DendriticNumber. |
| PH1-PH20 (20 traits) | N.A. | branching | topology | This is MDS1-MDS20 from a multidimensional scaling (MDS) on a pairwise bottleneck distance derived from persistence barcode data. |
| CC1-CC20 (20 traits) | N.A. | branching | topology | The count of connected components at specific geodesic distance threshold. This geodesic distance measures the shortest distance that travels along the phytolith surface to the core body. This trait is equivalent to count the number of bars at a specific value at x axis in persistence barcode. |
