# Supplementary material for: Elongate dendritic phytoliths as indicators for cereal identification and domestication: exploring a 3D morphometric approach
Source: Front Plant Sci. 2025 Oct 6;16:1643447. doi: 10.3389/fpls.2025.1643447 (PMC12535970; doi:10.3389/fpls.2025.1643447)
Supplement: Supplementary Table 3 — Morphometric trait reduction, component selection, and model evaluation for domestication and ploidy models. [file Table3.docx]

**SI Table 3: Morphometric trait reduction, component selection, and model evaluation for domestication and ploidy models**

| **Cluster** | **Variables_in_Cluster** | **Selected_Variable** |
| --- | --- | --- |
| 1 | Volume, CoreVolume, CoreConvexHullVolume, CoreSurfaceArea | Volume |
| 2 | ConvexHullVolume, SurfaceArea | ConvexHullVolume |
| 3 | Solidity | Solidity |
| 4 | Sphericity | Sphericity |
| 5 | MaxLength, PC1sd, CoreMaxLength, CorePC1sd | MaxLength |
| 6 | MaxWidth, PC2sd | MaxWidth |
| 7 | MaxHeight, PC3sd | MaxHeight |
| 8 | Elongation | Elongation |
| 9 | Flatness | Flatness |
| 10 | CoreOccupacity | CoreOccupancy |
| 11 | CoreSolidity | CoreSolidity |
| 12 | CoreSphericity, CoreElongation | CoreSphericity |
| 13 | CoreMaxWidth, CoreMaxHeight, CorePC2sd, CorePC3sd | CoreMaxWidth |
| 14 | CoreFlatness | CoreFlatness |
| 15 | DendriticNumber | DendriticNumber |
| 16 | DendriticDensity | DendriticDensity |
| 17 | DendriticTotalLength | DendriticTotalLength |
| 18 | AverageDendriticLength | AverageDendriticLength |
| 19 | CC_1, CC_2 | CC_1 |
| 20 | CC_3 | CC_3 |
| 21 | CC_4, CC_5, CC_6 | CC_4 |
| 22 | CC_7, CC_8 | CC_7 |
| 23 | CC_9, CC_10, CC_11 | CC_9 |
| 24 | CC_12, CC_13 | CC_12 |
| 25 | CC_14, CC_15 | CC_14 |
| 26 | CC_16, CC_17 | CC_16 |
| 27 | CC_18 | CC_18 |
| 28 | CC_19, CC_20 | CC_19 |

SI Table 3.1: Summary of trait clustering to reduce multicollinearity. Traits were grouped based on hierarchical clustering of pairwise absolute correlations (threshold > 0.9). For each cluster, one representative trait was selected for further analysis.

**SI Figure 3.2: Hierarchical clustering of highly correlated traits.**
Traits with a correlation above 0.9 were grouped into clusters for dimensionality reduction (see 7.1).

SI Figure 3.3: Scree plot showing the percentage of variance explained by each rotated component (RC) from the Domestication PCA with varimax rotation, based on mean morphometric values per sample. The plot indicates a gradual decline in explained variance, with the elbow between RC6 and RC7. Since this scree plot does not indicate a clear cut-off, we tested both models using both 6 and 7 RCs as input variables, selecting the 7-RC model due to its lower AIC (see below).

| Model | AIC | BIC |
| --- | --- | --- |
| 7 rotated components | 118.3357 | 138.4226 |
| 6 rotated components | 120.0903 | 137.6664 |

SI Table 3.4: **Comparison of logistic regression models predicting domestication status.** AIC and BIC values are reported for two models: a full model using 7 rotated components (RCs) and a reduced model using 6 rotated components (RCs).

Supplementary Data File 3.5: Heatmap showing the loadings of each trait on the rotated components in the domestication model. Warmer colors (red) indicate strong positive loadings, cooler colors (blue) indicate strong negative loadings, and white represents near-zero contributions. Traits are listed on the y-axis, and rotated components (RCs) are shown on the x-axis.

SI Figure 3.6: Scree plot showing the percentage of variance explained by each rotated component (RC) from the Ploidy level PCA with varimax rotation, based on mean morphometric values per sample. The plot indicates a gradual decline in explained variance, with the elbow at RC7.
