# Supplementary material for: Elongate dendritic phytoliths as indicators for cereal identification and domestication: exploring a 3D morphometric approach
Source: Front Plant Sci. 2025 Oct 6;16:1643447. doi: 10.3389/fpls.2025.1643447 (PMC12535970; doi:10.3389/fpls.2025.1643447)
Supplement: Supplementary Table 4 — Confusion matrices for lineage-, species-, and Triticum subspecies-level classifications for the test and test_other set, and output metrics for test_other set. [file DataSheet4.docx]

**SI Table 4: Confusion matrices for lineage-, species-, and *Triticum* subspecies-level classifications for the test and test_other set, and output metrics for test_other set.**

**SI Figure 4.1:** Confusion matrix for lineage-level classification on the test set. The heatmap shows the frequency of predicted versus actual lineage labels, with darker blue indicating higher classification counts. Labels on the diagonal represent correct classifications.

**SI Figure 4.2:** Confusion matrix for lineage-level classification on the test_other set. The heatmap shows the frequency of predicted versus actual lineage labels, with darker blue indicating higher classification counts. Labels on the diagonal represent correct classifications.

**SI Figure 4.3:** Confusion matrix for species-level classification on the test set. The heatmap shows the frequency of predicted versus actual species labels, with darker blue indicating higher classification counts. Labels on the diagonal represent correct classifications.

**SI Figure 4.4:** Confusion matrix for species-level classification on the test_other set. The heatmap shows the frequency of predicted versus actual species labels, with darker blue indicating higher classification counts. Labels on the diagonal represent correct classifications.

**SI Figure 4.5:** Confusion matrix for *Triticum* subspecies-level classification on the test set. The heatmap shows the frequency of predicted versus actual subspecies labels, with darker blue indicating higher classification counts. Labels on the diagonal represent correct classifications.

**SI Figure 4.6:** Confusion matrix for *Triticum* subspecies-level classification on the test set. The heatmap shows the frequency of predicted versus actual subspecies labels, with darker blue indicating higher classification counts. Labels on the diagonal represent correct classifications.

| **Dataset** | **Num_Classes** | **Num_Samples** | **Accuracy** | **Kappa** | **NIR** | **P_Value** |
| --- | --- | --- | --- | --- | --- | --- |
| Lineage | 1 | 287 | 0.53659 | 0 | 1 | 1 |
| Species | 18 | 991 | 0.09687 | 0.06161 | 0.20989 | 1 |
| *Triticum* subspecies | 8 | 170 | 0.15882 | 0.05611 | 0.14706 | 0.36412 |

**SI Table 4.7:** Classification performance on phytoliths from entirely unseen inflorescence samples (“test_other” set), representing taxa present in the training data but excluded from model fitting due to class imbalance. Metrics include the number of classes and samples evaluated, overall accuracy, Cohen’s Kappa, no-information rate (NIR), and p-value from a one-sided exact binomial test comparing observed accuracy to the NIR. A p-value close to 1 indicates that the model does not perform better than random guessing.
